# Molecular outcomes, clinical consequences, and genetic diagnosis of Oculocutaneous Albinism in Pakistani population

**DOI:** 10.1038/srep44185

**Published:** 2017-03-07

**Authors:** Mohsin Shahzad, Sairah Yousaf, Yar M. Waryah, Hadia Gul, Tasleem Kausar, Nabeela Tariq, Umair Mahmood, Muhammad Ali, Muzammil A. Khan, Ali M. Waryah, Rehan S. Shaikh, Saima Riazuddin, Zubair M. Ahmed, University of Washington Center for Mendelian Genomics (UW CMG) Consortium, Michael J. Bamshad, Jay Shendure, Deborah A. Nickerson, Gonçalo R. Abecasis, Peter Anderson, Elizabeth Marchani Blue, Marcus Annable, Brian L. Browning, Kati J. Buckingham, Christina Chen, Jennifer Chin, Jessica X. Chong, Gregory M. Cooper, Colleen P. Davis, Christopher Frazar, Tanya M. Harrell, Zongxiao He, Preti Jain, Gail P. Jarvik, Guillaume Jimenez, Eric Johanson, Goo Jun, Martin Kircher, Tom Kolar, Stephanie A. Krauter, Niklas Krumm, Suzanne M. Leal, Daniel Luksic, Colby T. Marvin, Sean McGee, Karynne Patterson, Marcos Perez, Sam W. Phillips, Jessica Pijoan, Christa Poel, Seamus Ragan, Frederic Reinier, Peggy D. Robertson, Regie Santos-Cortez, Aditi Shankar, Krystal Slattery, Cindy Shephard, Kathryn M. Shively, Deborah L. Siegel, Joshua D. Smith, Holly K. Tabor, Monica Tackett, Marc Wegener, Gao Wang, Marsha M. Wheeler, Amber Wright, Qian Yi

**Affiliations:** 1 Department of Otorhinolaryngology Head and Neck Surgery, School of Medicine, University of Maryland, Baltimore, MD, USA; 2 Institute of Molecular Biology & Biotechnology, Bahauddin Zakariya University, Multan, Pakistan; 3 Molecular Biology & Genetics Department, Medical Research Center, Liaquat University of Medical & Health Sciences, Jamshoro, Pakistan; 4 Gomal Centre of Biochemistry and Biotechnology, Gomal University, D.I. Khan, Pakistan; 5 Government College University, Faisalabad, Pakistan; 6 University of Washington, Seattle, WA, USA; 7 Seattle Children’s Hospital, Seattle, WA, USA; 8 University of Michigan, MI, USA; 9 Hudson Alpha Institute of Technology, AL, USA; 10 Baylor College of Medicine, TX, USA

## Abstract

Nonsyndromic oculocutaneous Albinism (nsOCA) is clinically characterized by the loss of pigmentation in the skin, hair, and iris. OCA is amongst the most common causes of vision impairment in children. To date, pathogenic variants in six genes have been identified in individuals with nsOCA. Here, we determined the identities, frequencies, and clinical consequences of *OCA* alleles in 94 previously unreported Pakistani families. Combination of Sanger and Exome sequencing revealed 38 alleles, including 22 novel variants, segregating with nsOCA phenotype in 80 families. Variants of *TYR* and *OCA2* genes were the most common cause of nsOCA, occurring in 43 and 30 families, respectively. Twenty-two novel variants include nine missense, four splice site, two non-sense, one insertion and six gross deletions. *In vitro* studies revealed retention of OCA proteins harboring novel missense alleles in the endoplasmic reticulum (ER) of transfected cells. Exon-trapping assays with constructs containing splice site alleles revealed errors in splicing. As eight alleles account for approximately 56% (95% CI: 46.52–65.24%) of nsOCA cases, primarily enrolled from Punjab province of Pakistan, hierarchical strategies for variant detection would be feasible and cost-efficient genetic tests for OCA in families with similar origin. Thus, we developed Tetra-primer ARMS assays for rapid, reliable, reproducible and economical screening of most of these common alleles.

Autosomal recessive non-syndromic oculocutaneous albinism (nsOCA) is a genetic disorder characterized by the partial or complete loss of pigmentation in the skin, hair, and iris that is due to a decrease or absence of melanin production^1^,^2^. Other clinically substantial aberrations associated with OCA are foveal hypoplasia, photoreceptor rod cell deficit, misrouting of the optic nerves at the chiasm, photophobia, nystagmus, and vision impairment^2^,^3^. The prevalence of albinism worldwide has been estimated at 1 in 17,000, meaning that approximately 1 in 70 people are carriers of the *OCA* allele. To date, pathogenic variants in six genes, *TYR, OCA2, TYRP1, SLC45A2, SLC24A5,* and *C10orf11*, have been identified in individuals with nsOCA^4^. We previously mapped a new locus for nsOCA on chromosome 4q24 in a large consanguineous Pakistani family^5^, for which the gene is currently unknown. In published population studies, however, the detection rate of alleles causing albinism varies from 60% to 90%^6^,^7^,^8^,^9^,^10^,^11^.

The clinical range of OCA varies, with OCA1A being the most severe type, characterized by a complete lack of melanin production throughout life. The less severe forms (OCA1B, OCA2, OCA3, and OCA4) show some pigmentation over a lifetime. Although the diverse types of OCA are caused by variants in different genes, their clinical phenotypes are not always distinguishable, making molecular diagnosis an essential tool for genetic counseling^12^, and for emerging therapeutic interventions^13^. Our goals here were to determine identities, frequencies, and clinical consequences of *OCA* alleles in Pakistani cohort, predominantly enrolled from Punjab province, and to develop hierarchical strategies for rapid, feasible, and cost-efficient genetic diagnostic assays for improved carrier detection and genetic counseling.

## Results

After institutional review board approval, we enrolled 94 Pakistani families segregating nsOCA (Supplementary Fig. S1). All the affected individuals of these families have congenital hypopigmentation phenotype. Inter-familial variation of hair color was noted among individuals, ranging from white to honey blonde or brown (Table 1). Similarly, variation in iris color was noted, with tones ranging from light grey to brown (Table 1). However, due to limited facilities available in the remote areas of Pakistan, detailed clinical evaluations in every affected person from every family was not possible, therefore, we refrained ourselves from commenting on genotype-phenotype correlation for every variant. Through the combination of Sanger and whole exome sequencing techniques, we identified 38 variants, including 22 novel variants, segregating with the phenotype in 80 families (Table 2). The 22 new variants include 9 missense, 4 splice site, 2 non-sense, 1 insertion, and 6 gross deletions (Table 2 and Fig. S2). None of these variants were found in ethnically matched control samples (Table 2 and Table S1). We also documented the frequencies of polymorphic alleles of ns*OCA* genes in our cohort (Table S2).

### Missense variants altered the targeting of encoded OCA proteins

The nine novel missense variants include two alleles of *TYR* (OCA1), six of *OCA2* (OCA2) and one in *TYRP1* (OCA3). Three prediction programs, specifically, Polyphen-2^14^, MutationTaster^15^ and SIFT^16^, suggested that each of these missense variants were deleterious (Table 2). To further evaluate the effects of these variants on the encoded proteins, we performed *in silico* molecular modeling and *in vitro* protein targeting studies.

**Figure 1:**
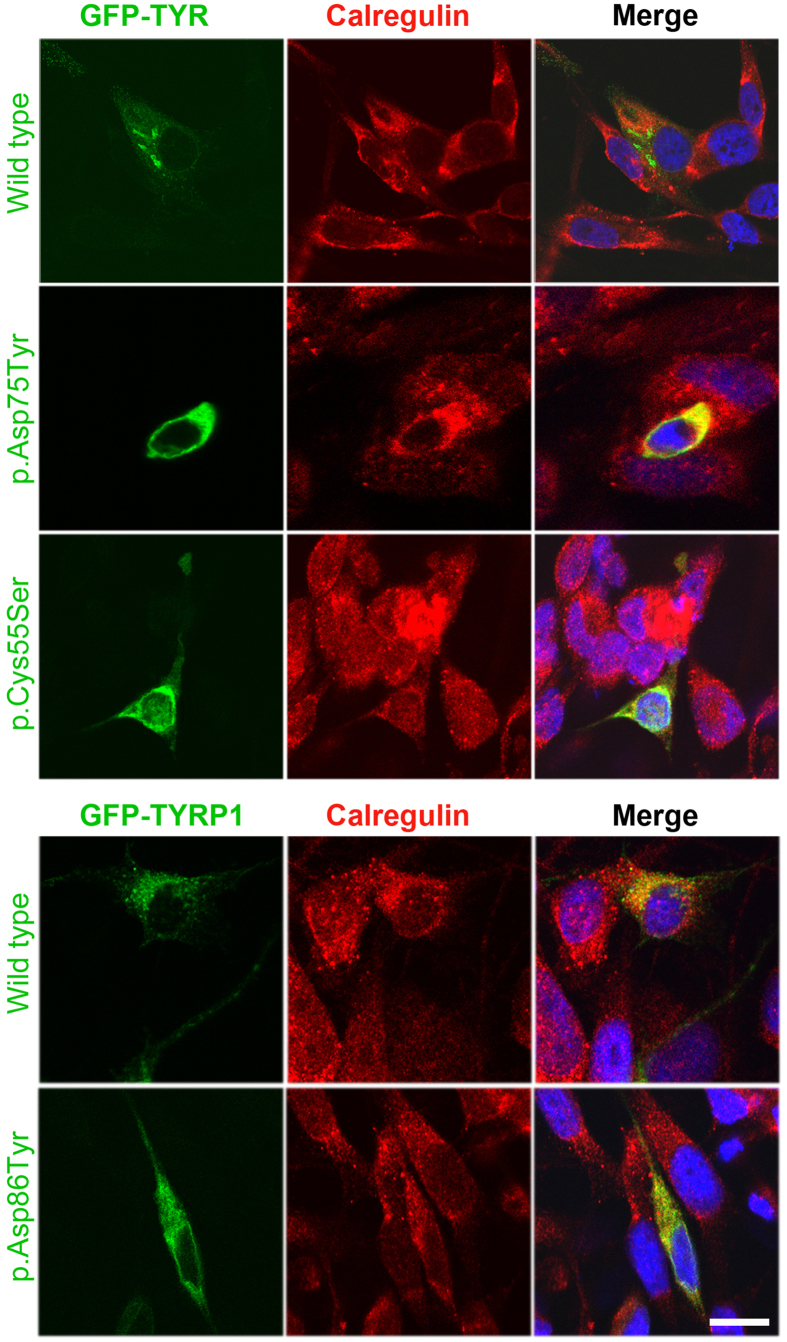
Subcellular distribution of wild type and mutant tyrosinase and tyrosinase-like proteins in human melanocytes. eGFP-tagged *TYR* and *TYRP1* wild type and mutant constructs (green) were transiently transfected in melanocytes grown at 37 °C. Calregulin (red) and DAPI (blue) were used as markers for the endoplasmic reticulum and nucleus, respectively. Merged images show the co-localization of only tyrosinase variants with calregulin, which indicated ER retention. The scale bar represents 20 μm for all panels.

*TYR* encodes tyrosinase in melanocytes, an essential enzyme for the biosynthesis of melanin^17^. Previously, it was shown that missense alleles of tyrosinase lead to ER retention of encoded protein due to misfolding^18^. To evaluate the targeting of p.Cys55Ser and p.Asp75Tyr harboring proteins, we introduced these variants in a GFP-tagged tyrosinase and transiently transfected human melanocytes. Wild type tyrosinase was localized throughout the cytoplasm of melanocytes (Fig. 1). Immunofluorescence studies with calregulin (an ER marker) demonstrated that the mutant proteins, however, predominantly co-localized with calregulin, indicating retention in the ER (Fig. 1). In contrast, p.(Asp86Tyr) missense variant did not affect the targeting of TYRP1 protein (Fig. 1).

**Figure 2:**
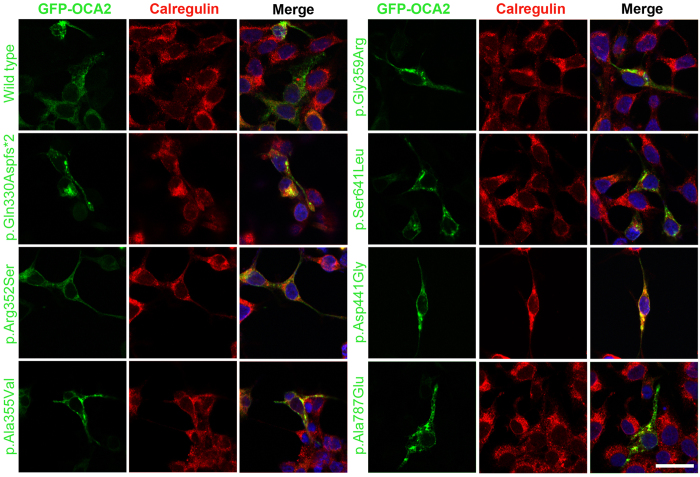
Subcellular distribution of wild type and mutant OCA2 in HEK293 cells. eGFP-tagged *OCA2* wild type and mutant constructs (green) were transiently transfected in HEK293 cells grown at 37 °C. Calregulin (red) and DAPI (blue) were used as markers for the endoplasmic reticulum and nucleus, respectively. Merged images show partial co-localization of OCA2 variants with calregulin, which indicated increased ER retention. The scale bar represents 20 μm for all panels.

OCA2 protein, with 12 putative transmembrane α-helices, belongs to the Na^+^/H^+^ antiporter family. Besides its presumed role in maintaining the pH of the melanosomes^19^,^20^,^21^,^22^, OCA2 also participates in the sorting and transport of tyrosinase and tyrosinase-related protein 1 (TYRP1) to the plasma membrane^23^,^24^,^25^. We performed comparative computational modeling of wild type and six novel missense alleles harboring OCA2 proteins using Phyre2^26^ program. All the identified missense alleles were predicted to impact either protein folding, interaction with the lipid-bilayer, protein topology, or protein-protein interactions (Fig. S3). When transiently transfected in HEK293 cells, in contrast to wild type, disease-associated alleles of OCA2 proteins showed retention in the ER (Fig. 2). Collectively, these studies support the deleterious nature of novel missense variants identified in our OCA cohort.

### *Ex vivo* splicing is defective due to splice site variants identified in Pakistani families

**Figure 3:**
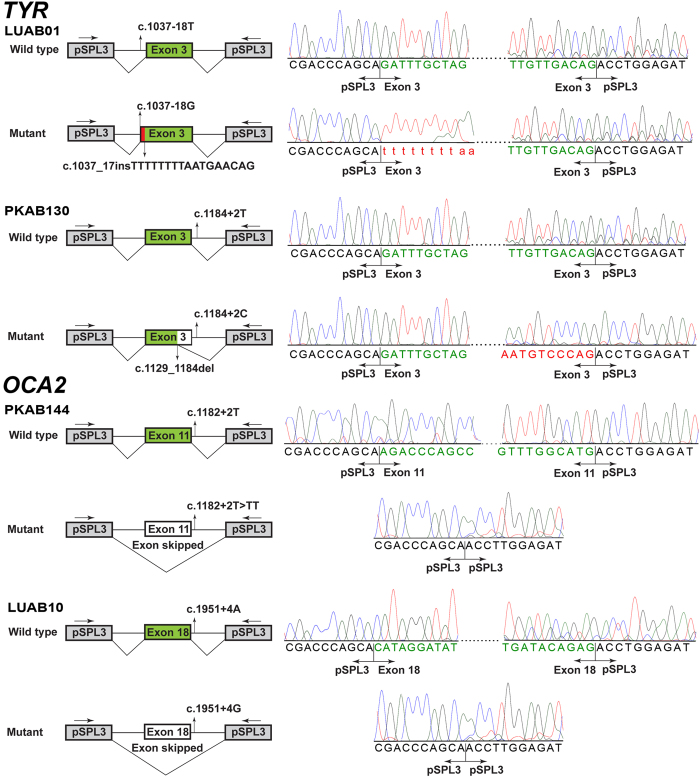
Functional analysis of splice site variants. Schematic representation and Sanger sequencing chromatograms of minigene assays for *TYR* and *OCA2* novel splice site variants revealed aberrant splicing products, supporting their pathogenic nature.

To evaluate the impact of four new splice site variants elucidated in our cohort, we examined the RNA splicing pattern of wild type and mutated exons by transfecting minigene constructs in COS7 cells. Results of our splicing assays are summarized in Fig. 3. The c.1037–18 T > G variant in exon 3 of *TYR* generated an upstream cryptic splice acceptor site, which resulted in insertion of 17 base intronic region in the spliced product (Fig. 3). Retention of 17 base intronic region in the spliced mRNA would result in the frameshift and premature truncation of the encoded tyrosinase. In contrast, the c.1184 + 2 T > C change in exon 3 of *TYR* revealed loss of canonical splice donor site. Splicing assay revealed utilization of a cryptic donor site within exon 3, resulting in loss of 55 bp from the coding region (Fig. 3), predicted to cause frameshift and premature truncation of the encoded protein. Both splice site variants of *OCA2* (c.1182 + 2 T > TT and c.1951 + 4 A > G) revealed skipping of their respective exons in the minigene splicing assays (Fig. 3). The skipping of exon 11 (66 bp) due to c.1182 + 2 T > TT leads to in-frame deletion of 22 amino acids, while the loss of exon 18 (109 bp) of *OCA2* due to c.1951 + 4 A > G variant would cause frameshift and premature truncation.

### Exome sequencing revealed six gross deletions in OCA families

**Figure 4:**
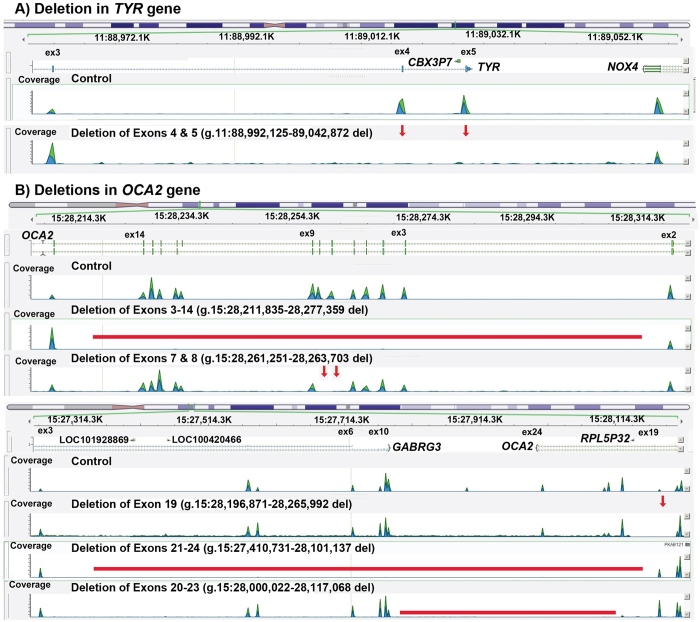
Gross genomic exonic deletions identified in six OCA families. (**A**) Compare the exome sequencing data (green-blue peaks) of control sample wild type sample, the exon 4 and 5 (indicated by red arrows) were deleted in DNA samples from PKAB64 and PKAB168 families. Breakpoints for genomic deletions are given according to the human genome build hg19/GRCh37. (**B**) Gross genomic deletions observed in *OCA2* gene as compared to control samples are shown either by red arrows or red lines.

Approaches to detect indels using exome sequence data are an active area of research. As yet there is no single method that guarantees consistent success. We used the widely-evaluated methods XHMM^27^ and CoNIFER^28^ to identify gross insertions/deletions in our exome data. Our analyses revealed six novel gross deletions of *TYR* and *OCA2* genes segregating with OCA phenotype in six families (Fig. 4). To investigate the mechanisms involved in these deletions, the intervals surrounding the breakpoints were analyzed through RepeatMasker (http://www.repeatmasker.org/). In addition, significantly over-represented motifs within ±15 bp of GRaBD translocations breakpoints were also sought^29^. Thorough bioinformatics analysis revealed that the breakpoint for these deletions lie in the repetitive element, and the highly similar *Alu* short interspersed nuclear elements (SINEs) may serve as the substrate for nonhomologous recombination.

Next, to analyze the impact of deletions on protein 3D structure, we used Phyre2 modeling software. Removal of p.Ser395 to p.Leu529 amino acids due to deletion of exons 4 and 5 would eliminate the tyrosinase central domain that binds to its copper ligand for subsequent function (Fig. S4A). Similarly, deletions of OCA2 exons would result in the partial or complete loss of Na-Sulphur-symporter domain, which mediates the intake of several different molecules with the concomitant uptake of Na^+^ (Fig. S4B) and thus predicted to result in non-functional, truncated proteins.

### Alleles of *TYR* and *OCA2* are the common cause of nsOCA in our cohort

**Figure 5:**
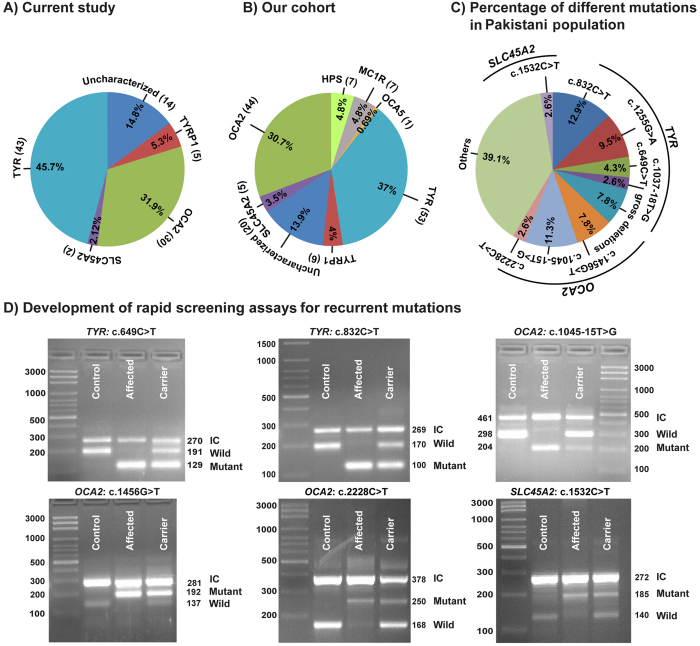
Prevalence of albinism genes and their alleles in a Pakistani population. (**A–C**) Relative distribution of variants in albinism genes in Pakistani families. (**A**) The distribution of OCA1-4 genes in 94 families screened in the current study. Number of families and their percentage contribution are given in parentheses. (**B**) Total pool of albinism genes in our cohort. (**C**) Frequencies of recurrent alleles of *nsOCA* genes in Pakistanis. (**D**) Results of tetra-primer ARMS assays for detection of recurrent variants. The top band in each gel represents the positive control amplimer in all samples generated using the outer primers. Nested allele-specific primers were used to generate the wild type (Wild) or variant harboring (Mutant) PCR products. IC: inner control product.

Variants of *TYR* (allele frequency: 15/38) and *OCA2* (19/38) were the most common cause of nsOCA, occurring in 43 and 30 families, respectively (Fig. 5A). To further refine the prevalence estimates, we reviewed the alleles frequencies in known albinism genes among our larger cohort of 143 families, including 48 previously reported families (Fig. 5B)^5^,^30^,^31^,^32^,^33^ and in published literature (Table S1). *TYR* and *OCA2* alleles are the frequent cause of OCA in Pakistanis (Table S1). In our cohort, variants in *TYR* and *OCA2* collectively account for the majority [67.8% (97/143); 95% confidence interval (CI): 60.2–76.0%] of the genetic causes of nsOCA, which is comparable to prevalence in European population (Table S3). In approximately 14% of our OCA families, we did not find any pathogenic variant in the known *OCA* genes (Fig. 5B).

Next, we investigated the frequency of alleles of *nsOCA* genes in our cohort. Overall, four alleles of *TYR*, three of *OCA2,* and one of *SLC45A2* together account for ~56% (95% CI: 46.52–65.24%) of the variants responsible for nsOCA in our cohort (Fig. 5C). Therefore, we developed rapid and inexpensive assays for detecting carriers and homozygotes (Fig. 5D). For most of these alleles, we were able to develop tetra-primer ARMS assays. The sensitivity and specificity of these assays were confirmed on multiple DNA samples with different genotypes followed by Sanger sequencing.

## Discussion

Our study illustrates the relative genetic contribution of four major *OCA* genes in the prevalence of albinism, primarily in families (69) currently residing in the Punjab province of Pakistan. However, our study also includes 19 families from Sindh and 6 families from Khyber Pakhtunkhwa (KPK) provinces. Pakistanis have a rich anthropogenic background owing to successive waves of invasions and adaptations of haplogroups. Most did not intermingle with the original local population and practiced endogamy, giving rise to genetic isolates that persists even today. Parental consanguinity is an important risk factor (0.25–20% higher chances) for recessive genetic defects^34^. In Pakistan, 62.7% of marriages are consanguineous, ~80% of which are between first cousins^35^. Specific clans and high consanguinity in Pakistan are the root causes of increased incidences of recessive disorders, including OCA. In our cohort, OCA phenotype was observed in families of different linguistic/ethnic origins (Table 1). We did not observe any apparent enrichment of a particular *OCA* allele within families of certain clans (Table 1). For instance, c.832 C > T in *TYR* and c.1045–15 T > G in *OCA2* are the most frequent alleles observed in our samples (Fig. 5C). However, both these alleles were observed in families of various ethnical and geographical origins (Table 1). Similarly, other common variants (e.g. c.649 C > T, c.1255 G > A, c.1456 G > T) were also found in families enrolled from Punjab, Sindh and KPK (Table 1). Therefore, with the current samples size for each of the identified allele, it would be inapplicable to comment on ethnic/linguistic origins of different alleles observed in our cohort.

Our overall estimated prevalence of *TYR* and *OCA2* alleles is quite similar in certain cases from Europe but fairly different from other studies in different populations (Table S3). For instance, *TYR* and *OCA2* variants account for 70% and 10%, respectively, of OCA in a study of 127 patients from a Chinese population (Table S3). In a few eastern and central regions of China, *TYR* and *OCA2* contribution varies; however, *TYR* alleles remain the most common cause of OCA. In India, a study of 82 OCA patients revealed approximately 60% and 11% prevalence for *TYR* and *OCA2*, respectively^36^. Similarly, in the US, Europe, Italy, Japan, and Korea, the alleles of *TYR* are the most common cause of nsOCA^7^,^8^,^9^,^11^,^37^ (Table S3). In contrast, variants in *OCA2* account for ~80% of the OCA cases in an African population^38^.

Usually the alleles of *TYR* result in the retention of encoded tyrosinase in the ER^18^,^30^. Here, we also observed retention of OCA2 proteins harboring missense and truncating alleles in the ER (Fig. 2). A portion of the known human and mouse *TYR* variants have shown temperature-sensitive behavior^30^,^39^,^40^,^41^,^42^,^43^,^44^. Cultivating mammalian cells at permissive temperature (31 °C) resulted in increased cytoplasmic expression of tyrosinase harboring missense alleles, especially for those present in the copper-binding region^30^,^39^,^41^,^42^,^44^. However, the new *OCA2* alleles were imaged at 31 °C and did not reveal an apparent increased cytoplasmic expression (data not shown).

Besides single nucleotide variants, our study, for the first time in Pakistani population, revealed six novel gross deletions in *TYR* and *OCA2* genes (Fig. 4). These genetic aberrations span from a single exon deletion up to 12 exons with the encompassed introns (Fig. 4). These deletions confer pigmentation disorders either because of almost entire protein would be missing or non-functional truncated forms would be encoded. Considering their relative contribution simplified methods and bioinformatics tools will be needed for rapid detection of gross deletions for clinical genetic diagnosis of OCA.

Clinical presentation of OCA is often not very helpful in genetic diagnosis due to significantly overlapping features (Table 1). For economic and geographical reasons, it is not feasible to routinely perform Sanger sequencing of all the known OCA genes to detect underlying genetic defects. However, PCR-based assays that are developed to detect common alleles are quick and affordable. Twenty of the variants identified in this study were private (found in single families). However, eight variants (Fig. 5C) account for more than half of the alleles found in our families (Table 2). Therefore, we developed tetra-primer ARMS assays for these common alleles for rapid detection and genetic screening in large cohorts before embarking for exome/genome wide studies. We are cognizant of the fact that the majority of families in our cohort are from the Punjab province of Pakistan. Hence, the details of a hierarchical nonsyndromic OCA genes mutational screening strategy may need to be refined for other geographical regions and lingo-ethnic groups within Pakistan. Our results will be helpful for future diagnosis, genetic counseling, molecular epidemiology, and functional studies of nsOCA genes associated pigmentation disorders.

Intriguingly, fourteen families in our cohort did not reveal any pathogenic variants in common *nsOCA* genes. There are several possibilities for our failure to detect disease-associated alleles in these fourteen families. First, potential pathogenic variants may alter sequence of cis-acting regulatory or deep intronic splicing elements that are necessary for expression of these genes in melanocytes. Presently, we have very limited knowledge of the location of the regulatory elements of *nsOCA* genes. Secondly, the hypopigmentation segregating in these fourteen families may be syndromic and requires comprehensive clinical phenotyping, which would help in filtering the candidate genetic variants for further assessments. Third, there may be additional genes responsible for OCA in humans. For instance, in the US population the detection rate of alleles in known *nsOCA* genes is around 75%^10^. Thus, there exists both a need for further genetic understanding of albinism and an opportunity to improve the molecular diagnosis of albinism, and quite possibly its prevention.

## Methods

### Patients

This study followed the tenets of the Declaration of Helsinki. This study was approved by the IRB Committees at the University of Maryland School of Medicine (UMSOM), USA, the Institute of Molecular Biology & Biotechnology, Multan, Liaquat University of Medical & Health Sciences, Jamshoro, and Gomal Centre of Biochemistry and Biotechnology, Gomal University, D.I. Khan, Pakistan. All the methods were performed in accordance with the UMSOM relevant guidelines and regulations. Pedigrees were drawn after interviewing multiple individuals to confirm the relationships. Informed written consents were obtained from the adult subjects and the parents of minor subjects. Two to five ml of peripheral blood samples were collected from each participating individual. Human genomic DNA was isolated from peripheral blood by using inorganic method^45^. Detailed medical histories were taken for all of the participating individuals of the enrolled families, including the hypopigmentation phenotype of the hair, skin, eye, disease onset, segregation, presence of eye abnormalities (nystagmus, strabismus, photophobia, and poor vision) and information about immunological, neurological or bleeding time.

### Sanger sequencing and segregation analysis

Primers (Table S4) covering the coding regions of the *TYR, OCA2, TYRP1* and *SLC45A2* genes were designed in primer3 software (http://bioinfo.ut.ee/primer3-0.4.0/primer3/input.htm) for Sanger sequencing and segregation analysis. PCR amplification, cleaning, and sequencing reactions were performed as previously described^30^,^32^.

### Exome Sequencing

DNA samples from some of the participating family members were submitted for exome sequencing (WES) at the Center for Mendelian Genomics (CMG), University of Washington. WES on PKAB137 was performed at the Genetic core facility of Cincinnati Children’s Hospital. Genomic libraries were recovered for exome enrichment using the NimbleGen Exome kits (Roche Diagnostics; San Francisco, CA). Paired-end sequencing was performed using the Illumina Hi-Seq 2000 system (Illumina, San Diego, CA). The obtained sequencing data were analyzed following the guidelines outlined in the Broad Institute’s Genome Analysis Toolkit^46^. The raw data were mapped using the Burrows Wheeler Aligner^47^. Variants were called using Unified Genotyper software, and the data were then subjected to further processing and quality control^46^,^47^. Golden Helix software was used to analyze the variants found through exome sequencing. The coverage of exome was checked through Golden Helix Genome Browser.

### Bioinformatics Analysis

Pathogenicity of novel missense variants was assessed by different bioinformatics prediction programs; Polyphen-2, Sift, and Mutation Taster. Allele frequencies of identified variants were checked in 1000 Genome browser (http://browser.1000genomes.org/), NHLBI-ESP variant database (http://evs.gs.washington.edu/EVS/) and ExAC database (http://exac.broadinstitute.org/). Multiple sequence alignments of orthologous OCA proteins were performed by using Clustal Omega multiple alignment program (http://www.ebi.ac.uk/Tools/msa/clustalo/). The 3D protein structures were predicted by Phyre2 server (http://www.sbg.bio.ic.ac.uk/phyre2/html/page.cgi?id=index) with “Intensive Mode” option which is a combination of template-based modeling and *ab initio* methods^48^.

### Tetra Primer Amplification Refractory Mutation System (ARMS) assay

PCR primers (Table S5) used for Tetra-primers ARMS assay were designed by adjusting maximum (1:8) and minimum (1:3) relative size difference of two inner products and keeping others by default settings. Reaction mixture contains 10 ul 2X ECONOTAQ, 0.5 ul of 10 Um outer primers and 1.0 ul of 10 Um inner primers each, 50 ng (2 ul) genomic DNA and 5 ul H_2_O. The thermo cycling conditions are: initial denaturation of 4 min at 95 °C and followed by 35 cycles of 95 °C for 30 sec, 60 °C for 45 sec, 72 °C for 45 sec, and a final extension at 72 °C for 8 min. The PCR product was visualized in 2.0% agarose gel stained with ethidium bromide.

### Exon-trapping assay

To ascertain the consequences of novel splice site variants found in our OCA families, the wild and mutant exons along with flanking intronic region (200 bp) were PCR amplified, cloned in pSPL3 vector (Invitrogen, Carlsbad, CA) and sequence verified as described^49^. Purified cloned constructs were transfected into COS7 cells using PEI (Polyethylenimine). After 48 hours of transfection, RNA was extracted using TRIzol reagent (Invitrogen, Carlsbad, CA) and single stranded cDNA (Clontech, Mountain View, CA) was synthesized. Primary PCR amplification of cDNA was performed using SD6 and SA2 vector primers and amplified products were cloned in TA-cloning vector (Invitrogen). At least ten bacterial clones for each construct were Sanger sequenced.

### Expression constructs, transfection and immunofluorescence

All expression constructs used for immunofluorescence study were in eGFP-tagged vector (Clontech). For generating mutant constructs, mutagenesis was performed by using QuikChange kit (Stratagene, La Jolla, CA) and used specific wild type eGFP construct as a template. Each construct was transiently transfected in melanocytes or HEK293 cells seeded on cover slips with Lipofectamine 3000 (Invitrogen). After 48 hours of transfection either at 37 °C or 31 °C, cells were fixed and permeabilized in 4% paraformaldehyde and 0.1% Triton X-100 in PBS, respectively. For endoplasmic reticulum and nucleus visualization anti-Calregulin antibodody (Santa Cruz Biotechnology, Santa Cruz, CA) and DAPI staining was performed. A Zeiss LSMDUO confocal microscope was used for imaging.

## Additional Information

**How to cite this article:** Shahzad, M. *et al*. Molecular outcomes, clinical consequences, and genetic diagnosis of Oculocutaneous Albinism in Pakistani population. *Sci. Rep.* **7**, 44185; doi: 10.1038/srep44185 (2017).

**Publisher's note:** Springer Nature remains neutral with regard to jurisdictional claims in published maps and institutional affiliations.

## Supplementary Material

Supplementary Information

## Figures and Tables

**Table 1:** Clinical features of oldest affected individuals of families with variants in OCA1-4.

Families	ID	Sex	Allele	Ethnicity	Skin color	Hair color	Iris color	Visual Acuity		Type of refraction error	Fundus	Foveal hypoplasia	Photophobia	Nystagmus	
								Right Left	Right Left						
***TYR***															
PKAB115	IV:4	M	c.164 G > C	Jatt sidhu	White	Yellow white	Grey blue	NA	NA	NA	NA	NA	NA	Negative	
PKAB174	IV:4	M	c.223 G > T	Qureshi	White	Yellow white	Grey blue	NA	NA	NA	NA	NA	NA	Positive	
PKAB200	IV:4	M	c.585 G > A	Gondal	White	White	Grey blue	NA	NA	NA	NA	NA	NA	Negative	
PKAB66	V:3	M	c.649 C > T	Alvi	White	Yellow white	Brown	NA	NA	NA	NA	NA	NA	Positive	
PKAB76	IV:7	M	c.649 C > T	Mochi	White	Yellow white	Brown	NA	NA	NA	NA	NA	NA	Positive	
PKAB141	IV:3	M	c.649 C > T	Arain	White	Yellow white	Hazel green	NA	NA	NA	NA	NA	NA	Positive	
PKAB142	IV:3	M	c.649 C > T	Minhas	White	Yellow white	Hazel green	NA	NA	NA	NA	NA	NA	Positive	
PKAB147	III:3	M	c.649 C > T	Raajpoot	White	Yellow white	Hazel green	NA	NA	NA	NA	NA	NA	Positive	
PKAB118	IV:2	F	c.832 C > T	Mula khel	White	White	Grey blue	6/12	6/12	−1.00/-2.5*160–1.00/-1.00*20	Albinotic	Positive	Positive	Positive	
PKAB120	III:13	M	c.832 C > T	Arain	White	White	Grey	6/60	6/60	−4.00*180–3.50*180	Albinotic	Positive	Positive	Positive	
PKAB143	IV:7	M	c.832 C > T	Malick	White	White	Grey	6/60	6/60	Hyperopic	Albinotic	Positive	Positive	Positive	
PKAB145	IV:2	F	c.832 C > T	Arain	White	Yellow white	Grey blue	NA	NA	NA	NA	NA	NA		
PKAB165	IV:8	M	c.832 C > T	Jatt	White	White	Grey blue	6/60	6/60	Hyperopic	Albinotic	Positive	Positive	Negative	
PKAB183	VI:1	F	c.1037–7 T > A	Sayyad bukhari	Yellow	Pinkish	Brownish	6/60	6/60	Hyperopic	Albinotic	Positive	Positive	Positive	
PKAB185	IV:4	F	c.832 C > T	Sayyad bukhari	White	White	Grey blue	6/60	6/60	Hyperopic	Albinotic	Positive	Positive	Positive	
PKAB186	IV:3	F	c.1037–7 T > A	Sayyad bukhari	Yellow	Pinkish	Brownish	6/60	6/60	Hyperopic	Albinotic	Positive	Positive	Positive	
PKAB188	VI:1	M	c.1037–7 T > A	Khokar	Yellow	Pinkish	Brownish	6/60	6/60	Hyperopic	Albinotic	Positive	Positive	Positive	
PKAB189	V:11	M	c.832 C > T	Bhatti	White	White	Grey blue	NA	NA	NA	NA	NA	NA	NA	
PKAB194	VI:2	M	c.832 C > T	Raajpoot	White	White	Grey blue	NA	NA	NA	NA	NA	NA	NA	
LUAB05	IV:8	F	c.832 C > T	Urdu speaking	Pinkish white	White	Grey blue	6/60	6/60	Myopic type	Pigmented	Poorly formed	Positive	Positive	
LUAB08	V:7	M	c.832 C > T	Urdu speaking	White	White	Grey blue	4/60	4/60	Clear	Absent	Positive	Positive	Negative	
LUAB19	IV:3	M	c.832 C > T	Urdu speaking	White	White	Grey blue	NA	NA	NA	NA	NA	NA	NA	
PKAB204	IV:9	M	c.832 C > T	Jatt	White	White	Grey blue	NA	NA	NA	NA	NA	NA	Positive	
PKAB128	IV:3	M	c.896 G > A	Abbasi	White	White	Grey blue	NA	NA	NA	NA	NA	NA	NA	
PKAB154	IV:5	M	c.896 G > A	Waseer	White	White	Grey blue	NA	NA	NA	NA	NA	NA	NA	
PKAB138	III:7	F	c.943–948delTCAGCT	Butt	White	White	Grey blue	6/12	6/18	−3.00*20–0.50/-2.00*160	Albinotic	Positive		Positive	
LUAB01	V:4	M	c.1037–18 T > G	Urdu speaking	White	White	Light brown	6/60	6/60	Hypermetropia	Clear	Poorly formed	Positive	Positive	
PKAB105	IV:9	M	c.1037–7 T > A	Gouraya	White	Yellow white	NA	NA	NA	NA	NA	NA	NA	Negative	
PKAB197	IV:3	M	c.1037 G > A	Sahoo	White	White	Grey blue	NA	NA	NA	NA	NA	NA	NA	
PKAB119	IV:9	M	c.1147 G > A	Bangulzai	White	White	Grey/brown	6/12	6/18	+ 1.50/-3.00*10 + 1.50/-3.00*180	Albinotic	Positive	Positive	Positive	
PKAB130	IV:8	F	c.1184 + 2 T > C	Bhatti	White	White	Light green	6/60	6/60	Myopic astigmatism	Albinotic	Positive	Positive	Positive	
PKAB193	IV:2	F	c.1204 C > T	Gujar	White	White	Grey blue	NA	NA	NA	NA	NA	NA	NA	
PKAB64	III:6	M	Exons 4–5 deletion	Jatt	Golden	Blonde	Brownish green	6/60	6/60	+ 1.75/0*160–2.75/-2*180	—	Present	Positive	Present	
PKAB168	IV:8	M	Exons 4–5 deletion	Chakotaray	Grey white	White	Brown	NA	NA	NA	NA	Positive	Positive	Positive	
PKAB81	IV:5	F	c.1255 G > A	Rajpoot	White	Yellow white	Grey	6/60	6/24	Hyperopic and astigmatic	Albinotic	Positive	Positive	Positive	
PKAB106	IV:4	M	c.1255 G > A	Shaikh	White	Golden yellow	Grey blue	NA	NA	NA	NA	NA	NA	NA	
PKAB107	IV:1	F	c.1255 G > A	Chanarr	White	Yellow white	Grey blue	6/60	6/60	Hyperopic	Albinotic	Positive	Positive	Positive	
PKAB140	IV:7	F	c.1255 G > A	Daaye	White	Yellow white	Grey blue	6/18	6/18	Myopic	Albinotic	Positive	Positive	Positive	
PKAB184	IV:6	M	c.1255 G > A	Oteera	White	White	Grey blue	CF	HMt	NA	Albinotic	Positive	Positive	Positive	
PKAB191	V:4	M	c.1255 G > A	Dodiyaanay Sial	White	White	Grey blue	NA	NA	NA	NA	NA	NA	NA	
LUAB03	V:6	M	c.1255 G > A	Sindhi speaking	Pinkish white	White	Hazel	1/60	1/60	No improvement	Clear	Poorly formed	Positive	Positive	
GUAB01	IV:6	M	c.1255 G > A	Saraiki speaking	Light blonde	Light blonde	Blue grey	NA	NA	NA	NA	NA	Positive	Positive	
***OCA2***															
PKAB70	IV:7	M	Exons 3–14 deletion	Maachi	White	Yellow	Green	6/36	6/36	Hyperopic	Albinotic	Positive	Positive	Positive	
PKAB69	V:6	M	Exons 7–8 deletion	Mughal	Pink white	Silver yellow	Grey	CF	CF	−3.00/-3.50*178–3.00/-4.00*3	Albinotic	Positive	Positive	Positive	
PKAB77	IV:6	M	Exons 7–8 deletion	Terkhan	Pink white	Silver Yellow	Grey	6/36	6/36	−1.75/-1.005–5.25/-1.25*20	Albinotic	Positive	Positive	Positive	
PKAB127	IV:4	M	c.1045–15 T > G	Rajpoot	White	Yellow	Hazel brown	6/48	6/48	Compound astigmatism	Albinotic	Positive	Positive	Positive	
PKAB132	III:5	M	c.1045–15 T > G	Jatt	White	Yellow white	Light green	NA	NA	NA	NA	NA	NA	NA	
PKAB134	III:3	M	c.1045–15 T > G	Abbasi	White	Yellow white	Light green	NA	NA	NA	NA	NA	NA	NA	
PKAB135	IV:3	M	c.1045–15 T > G	Arain	White	White	Light green	NA	NA	NA	NA	NA	NA	NA	
PKAB177	IV:7	F	c.1045–15 T > G	Bhatti	White	Yellow	Hazel brown	6/48	6/48	Compound astigmatism	Albinotic	Positive	Positive	Positive	
GUAB03	IV:8	F	c.1045–15 T > G	Saraiki speaking	Brown	Brown	Greenish	NA	NA	NA	NA	NA	Positive	Positive	
LUAB07	IV:3	M	c.1045–15 T > G	Sindhi speaking	White	White	Light brown	6/18	6/18	Hypermetropia	Pigmented	Poorly formed	Positive	Positive	
LUAB02	IV:8	M	c.1056 A > C	Sindhi speaking	White	White	Light brown	3/18	3/18	Hypermetropia	Pigmented	Poorly formed	Positive	Positive	
PKAB136	IV:3	F	c.1064 C > T	Chandia	White	Yellow white	Light green	NA	NA	NA	NA	NA	NA	NA	
GUAB02	IV:6	M	c.1075 G > C	Saraiki speaking	Light golden	Light golden	Greenish	NA	NA	NA	NA	NA	Positive	Positive	
PKAB144	IV:2	M	c.1182 + 2 T > TT	Arain	White	Yellow white	Grey blue	NA	NA	NA	NA	NA	NA	NA	
PKAB148	IV:5	M	c.1211 C > T	Sayyad	White	Golden yellow	Grey blue	NA	NA	NA	NA	NA	NA	NA	
LUAB06	V:8	M	c.1322 A > G	Sindhi speaking	Pinkish white	Golden white	Light brown	6/60	6/60	Mycopic Type	Pigmented	Poorly formed	Positive	Positive	
PKAB172	V:1	F	c.1456 G > T	Wains	White	Yellowish brown	Brown	6/48	6/40	Myopic astigmatism	Albinotic	Positive	Positive	Positive	
PKAB187	III:3	M	c.1456 G > T	Bhutta	White	Golden yellow	Grey blue	NA	NA	NA	NA	NA	NA	NA	
GUAB06	IV:5	M	c.1456 G > T	Saraiki speaking	Golden	Golden	Greenish	NA	NA	NA	NA	NA	Positive	Positive	
LUAB17	IV:3	F	c.1456 G > T	Sindhi speaking	White	Yellow white	Grey blue	NA	NA	NA	NA	NA	NA	NA	
PKAB122	IV:2	F	c.1922 C > T	Mughal	White	Golden yellow	Light brown	6/24	6/24	NA	Albinotic	Positive	Positive	Positive	
LUAB10	III:9	F	c.1951 + 4 A > G	Urdu speaking	White	White	Light brown	6/18	6/18	Hypermetropia	Pigmented	Poorly formed	Positive	Positive	
PKAB137	IV:4	M	Exon 19 deletion	Malick	White	Yellow white	Hazel green	NA	NA	NA	NA	NA	NA	Positive	
PKAB121	IV:3	F	Exons 20–24 deletion	Achakzai	White	White	Brown	6/18	6/18	2.50*20–2.0*160	Albinotic	Positive	Positive	Negative	
PKAB116	IV:4	M	Exons 22–24 deletion	Raajpoot	White	Golden yellow	Misty grey	6/36	6/36	+3.75/-4.75*15 +1.75/+3.0*80	Albinotic	Positive	Positive	Positive	
LUAB09	IV:5	F	c.2228 C > T	Urdu speaking	White	Goldish white	Light brown	6/18	6/18	Hypermetropia	Pigmented	Poorly formed	Positive	Positive	
PKAB170	V:3	F	c.2360 C > A	Jatt Langa	White	Yellow	Brown	6/36	6/36	Hyperopic	Albinotic	Positive	Positive	Positive	
PKAB125	IV:5	M	c.2360 C > T	Arain	White	Golden yellow	NA	NA	NA	NA	NA	NA	NA	Positive	
***TYRP1***															
PKAB166	IV:3	F	c.256 G > T	Bhutta	Brown	Wheat	Brown	6/6	6/6	Emmetropic	Normal	Negative	Negative	Positive	
PKAB164	IV:5	M	c.1067 G > A	Langa	Grey brown	Wheat	Brown	6/24	6/24	−2.25/−5.25*97–0.50/−5.00*90	Albinotic	Positive	Positive	Positive	
PKAB192	V:2	M	c.1067 G > A	Haraaj	Wheat	Golden yellow	Light green	NA	NA	NA	NA	NA	NA	Positive	
PKAB205	IV:12	F	c.1067 G > A	Matam	Wheat	Golden yellow	Light green	NA	NA	NA	NA	NA	NA	Positive	
PKAB146	III:1	M	c.1532 C > T	Shaikh	Wheat	Yellow brown	Grey	6/9 P	6/9 P	+8.0/−1.0*10 +7.50/−1.00*180	Normal	Negative	Positive	Positive	
***SLC45A2***															
GUAB04	IV:5	M	c.1532 C > T	Saraiki speaking	Light brown	Golden	Bluish	NA	NA	NA	NA	NA	Positive	Positive	
GUAB05	II:9	M	c.1532 C > T	Saraiki speaking	Light brown	Golden	Bluish	NA	NA	NA	NA	NA	Positive	Positive	

NA: Not available.

**Table 2:** Overview of molecular outcome and predictive effects of alleles in *TYR, OCA2, TYRP1* and *SLC45A2* genes.

Families	Variant	Effect on protein	Allele frequency				Polyphen-2	Mutation Taster	SIFT
			PK	1000 genome	NHLBI10^−4^	ExAC 10^−6^			
***TYR***									
PKAB115	c.164 G > C	p.(Cys55Ser)	0/180	0.000	0	8.2	Probably damaging	Disease causing	Damaging
PKAB174	c.223 G > T	p.(Asp75Tyr)	0/176	0	0	0	Probably damaging	Disease causing	Damaging
PKAB200	c.585 G > A	p.(Trp195*)	0/186	0	1.53	8.2	—	—	—
PKAB66, PKAB76, PKAB141, PKAB142, PKAB147	c.649 C > T	p.(Arg217Trp)	—	0	1.53	197.9	Probably damaging	Polymorphism	Tolerated
PKAB118, PKAB120, PKAB143, PKAB145, PKAB165, PKAB185, PKAB189, PKAB194, LUAB05, LUAB08, LUAB19, PKAB204	c.832 C > T	p.(Arg278*)	—	0	0	190.0	—	—	—
PKAB128, PKAB154	c.896 G > A	p.(Arg299His)	—	0	0	74.2	Probably damaging	Disease causing	Damaging
PKAB138	c.943–948delTCAGCT	p.(315–316delSerAla)	—	0	0	0	—	—	—
PKAB105, PKAB183, PKAB186, PKAB188	c.1037–7 T > A	Splicing error	—	0	15.38	0	—	—	—
LUAB01	c.1037–18 T > G	Splicing error	0/176	0	0	16.7	—	—	—
PKAB197	c.1037 G > A	p.(Gly346Glu)		0					
PKAB119	c.1147 G > A	p.(Asp383Asn)	—	0	0	165	Probably damaging	Disease causing	Damaging
PKAB130	c.1184 + 2 T > C	Splicing error	0/178	0	0	8.2	—	—	—
PKAB193	c.1204 C > T	p.(Arg402*)	—	0	0	49.84	—	—	—
PKAB64, PKAB168	Exons 4–5 deletion	Frame shift	0/180	0	0		—	—	—
PKAB81, PKAB106, PKAB107, PKAB140, PKAB184, PKAB191, LUAB03, LUAB19, GUAB01	c.1255 G > A	p.(Gly419Arg)		0	0	33.12	Probably damaging	Disease causing	Damaging
***OCA2***									
PKAB070	Exons 3–14 deletion	Frame shift	0/176	0	0	0	—	—	—
PKAB069, PKAB77	Exons 7–8 deletion	Frame shift	0/180	0	0	0	—	—	—
LUAB04	c.987 C > AGA	p.(Gln330Aspfs*2)	0/184	0	0	0	—	—	—
PKAB127, PKAB132, PKAB133, PKAB134, PKAB135, PKAB177, LUAB07, GUAB03	c.1045–15 T > G	Splicing error		0		41.36	—	—	—
LUAB02	c.1056 A > C	p.(Arg352Ser)	0/182	0	0	0	Probably damaging	Disease causing	Damaging
PKAB136	c.1064 C > T	p.(Ala355Val)	0/184	0.001	0	289.2	Probably damaging	Disease causing	Tolerated
GUAB02	c.1075 G > C	p.(Gly359Arg)	0/176	0	0	0	Probably damaging	Disease causing	Damaging
PKAB144	c.1182 + 2 T > TT	Splicing error	0/180	0	0	0	—	—	—
PKAB148	c.1211 C > T	p.(Thr404Met)	—	0	7.69	74.17	Probably damaging	Disease causing	Damaging
LUAB06	c.1322 A > G	p.(Asp441Gly)	0/178	0	1.53	16.74	Probably damaging	Disease causing	Tolerated
PKAB172, PKAB187, GUAB06, LUAB17	c.1456 G > T	p.(Asp486Tyr)	—	0	0	16.74	Probably damaging	Disease causing	Damaging
PKAB122	c.1922 C > T	p.(Ser641Leu)	0/184	0	0	0	Probably damaging	Disease causing	Damaging
LUAB10	c.1951 + 4 A > G	Splicing error		0	0	0	—	—	—
PKAB137	Exon 19 deletion	Frame shift	0/174	0	0	0	—	—	—
PKAB121	Exons 20–24 deletion	Frame shift	0/176	0	0	0	—	—	—
PKAB116	Exons 22–24 deletion	Frame shift	0/180	0	0	0	—	—	—
LUAB09	c.2228 C > T	p.(Pro743Leu)	—	0	0	90.78	Probably damaging	Disease causing	Damaging
PKAB170	c.2360 C > A	p.(Ala787Glu)	0/182	0	0	0	Probably damaging	Disease causing	Damaging
PKAB125	c.2360 C > T	p.(Ala787Val)	—	0	0	24.78	Probably damaging	Disease causing	Damaging
*TYRP1*									
PKAB166	c.256 G > T	p.(Asp86Tyr)	0/180	0	0	0	Probably damaging	Disease causing	Damaging
PKAB164, PKAB192, PKAB205	c.1067 G > A	p.(Arg356Gln)	—	0	0	25.50	Probably damaging	Disease causing	Damaging
PKAB146	c.1534 C > T	p.(Glu512*)	0/184	0	0	16.53	—	—	—
***SLC45A2***									
GUAB04, GUAB05	c.1532 C > T	p.(Ala511Val)	—	0	0	8.2	Probably damaging	Disease causing	Damaging

Novel mutations found in this study are in bold. PK (Pakistani Control).

## References

[b1] Spritz R. A., Chiang P. W., Oiso N. & Alkhateeb A. Human and mouse disorders of pigmentation. Curr Opin Genet Dev 13, 284–289 (2003). 12787791 10.1016/s0959-437x(03)00059-5

[b2] Tomita Y. & Suzuki T. Genetics of pigmentary disorders. Am J Med Genet C Semin Med Genet 131C, 75–81, doi: 10.1002/ajmg.c.30036 (2004). 15452859

[b3] Oetting W. S. & King R. A. Molecular basis of albinism: mutations and polymorphisms of pigmentation genes associated with albinism. Hum Mutat 13, 99–115, doi: 10.1002/(SICI)1098-1004(1999)13:2<99::AID-HUMU2>3.0.CO;2-C (1999). 10094567

[b4] Montoliu L. . Increasing the complexity: new genes and new types of albinism. Pigment Cell Melanoma Res 27, 11–18, doi: 10.1111/pcmr.12167 (2014). 24066960

[b5] Kausar T., Bhatti M. A., Ali M., Shaikh R. S. & Ahmed Z. M. OCA5, a novel locus for non-syndromic oculocutaneous albinism, maps to chromosome 4q24. Clin Genet 84, 91–93, doi: 10.1111/cge.12019 (2013). 23050561

[b6] Hutton S. M. & Spritz R. A. A comprehensive genetic study of autosomal recessive ocular albinism in Caucasian patients. Invest Ophthalmol Vis Sci 49, 868–872, doi: 10.1167/iovs.07-0791 (2008). 18326704

[b7] Mauri L. . Clinical evaluation and molecular screening of a large consecutive series of albino patients. J Hum Genet, doi: 10.1038/jhg.2016.123 (2016). 27734839

[b8] Park S. H., Chae H., Kim Y. & Kim M. Molecular analysis of Korean patients with oculocutaneous albinism. Jpn J Ophthalmol 56, 98–103, doi: 10.1007/s10384-011-0098-z (2012). 22042571

[b9] Rooryck C. . Molecular diagnosis of oculocutaneous albinism: new mutations in the OCA1-4 genes and practical aspects. Pigment Cell Melanoma Res 21, 583–587, doi: 10.1111/j.1755-148X.2008.00496.x (2008). 18821858

[b10] Simeonov D. R. . DNA variations in oculocutaneous albinism: an updated mutation list and current outstanding issues in molecular diagnostics. Hum Mutat 34, 827–835, doi: 10.1002/humu.22315 (2013). 23504663 PMC3959784

[b11] Tomita Y., Miyamura Y., Kono M., Nakamura R. & Matsunaga J. Molecular bases of congenital hypopigmentary disorders in humans and oculocutaneous albinism 1 in Japan. Pigment Cell Res 13 Suppl 8, 130–134 (2000). 11041370 10.1034/j.1600-0749.13.s8.23.x

[b12] Oetting W. S., Fryer J. P., Shriram S. & King R. A. Oculocutaneous albinism type 1: the last 100 years. Pigment Cell Res 16, 307–311 (2003). 12753405 10.1034/j.1600-0749.2003.00045.x

[b13] Onojafe I. F. . Nitisinone improves eye and skin pigmentation defects in a mouse model of oculocutaneous albinism. J Clin Invest 121, 3914–3923, doi: 10.1172/JCI59372 (2011). 21968110 PMC3223618

[b14] Adzhubei I. A. . A method and server for predicting damaging missense mutations. Nat Methods 7, 248–249, doi: 10.1038/nmeth0410-248 (2010). 20354512 PMC2855889

[b15] Schwarz J. M., Rodelsperger C., Schuelke M. & Seelow D. MutationTaster evaluates disease-causing potential of sequence alterations. Nat Methods 7, 575–576, doi: 10.1038/nmeth0810-575 (2010). 20676075

[b16] Kumar P., Henikoff S. & Ng P. C. Predicting the effects of coding non-synonymous variants on protein function using the SIFT algorithm. Nat Protoc 4, 1073–1081, doi: 10.1038/nprot.2009.86 (2009). 19561590

[b17] Tripathi R. K., Hearing V. J., Urabe K., Aroca P. & Spritz R. A. Mutational mapping of the catalytic activities of human tyrosinase. J Biol Chem 267, 23707–23712 (1992). 1429711

[b18] Halaban R. . Endoplasmic reticulum retention is a common defect associated with tyrosinase-negative albinism. Proc Natl Acad Sci USA 97, 5889–5894 (2000). 10823941 10.1073/pnas.97.11.5889 PMC18529

[b19] Puri N., Gardner J. M. & Brilliant M. H. Aberrant pH of melanosomes in pink-eyed dilution (p) mutant melanocytes. J Invest Dermatol 115, 607–613, doi: 10.1046/j.1523-1747.2000.00108.x (2000). 10998131

[b20] Preising M. N. . Mutation analysis in a family with oculocutaneous albinism manifesting in the same generation of three branches. Mol Vis 13, 1851–1855 (2007). 17960121

[b21] Matsunaga J. . Sequence-based diagnosis of tyrosinase-related oculocutaneous albinism: successful sequence analysis of the tyrosinase gene from blood spots dried on filter paper. Dermatology 196, 189–193 (1998). 9568405 10.1159/000017897

[b22] Gershoni-Baruch R. . Mutations of the tyrosinase gene in patients with oculocutaneous albinism from various ethnic groups in Israel. Am J Hum Genet 54, 586–594 (1994). 8128955 PMC1918101

[b23] Rosemblat S. . Melanosomal defects in melanocytes from mice lacking expression of the pink-eyed dilution gene: correction by culture in the presence of excess tyrosine. Exp Cell Res 239, 344–352, doi: 10.1006/excr.1997.3901 (1998). 9521852

[b24] Orlow S. J. & Brilliant M. H. The pink-eyed dilution locus controls the biogenesis of melanosomes and levels of melanosomal proteins in the eye. Exp Eye Res 68, 147–154, doi: 10.1006/exer.1998.0599 (1999). 10068480

[b25] Chen K., Manga P. & Orlow S. J. Pink-eyed dilution protein controls the processing of tyrosinase. Mol Biol Cell 13, 1953–1964, doi: 10.1091/mbc.02-02-0022. (2002). 12058062 PMC117617

[b26] Kelley L. A., Mezulis S., Yates C. M., Wass M. N. & Sternberg M. J. The Phyre2 web portal for protein modeling, prediction and analysis. Nat Protoc 10, 845–858, doi: 10.1038/nprot.2015.053 (2015). 25950237 PMC5298202

[b27] Plagnol V. . A robust model for read count data in exome sequencing experiments and implications for copy number variant calling. Bioinformatics 28, 2747–2754, doi: 10.1093/bioinformatics/bts526 (2012). 22942019 PMC3476336

[b28] Krumm N. . Copy number variation detection and genotyping from exome sequence data. Genome Res 22, 1525–1532, doi: 10.1101/gr.138115.112 (2012). 22585873 PMC3409265

[b29] Chuzhanova N., Abeysinghe S. S., Krawczak M. & Cooper D. N. Translocation and gross deletion breakpoints in human inherited disease and cancer II: Potential involvement of repetitive sequence elements in secondary structure formation between DNA ends. Hum Mutat 22, 245–251, doi: 10.1002/humu.10253 (2003). 12938089

[b30] Jaworek T. J. . Molecular genetic studies and delineation of the oculocutaneous albinism phenotype in the Pakistani population. Orphanet J Rare Dis 7, 44, doi: 10.1186/1750-1172-7-44 (2012). 22734612 PMC3537634

[b31] Kausar T. . Genetic studies of TYRP1 and SLC45A2 in Pakistani patients with nonsyndromic oculocutaneous albinism. J Invest Dermatol 133, 1099–1102, doi: 10.1038/jid.2012.432 (2013). 23190901

[b32] Shahzad M. . Identification and functional characterization of natural human melanocortin 1 receptor mutant alleles in Pakistani population. Pigment Cell Melanoma Res 28, 730–735, doi: 10.1111/pcmr.12400 (2015). 26197705 PMC4609612

[b33] Yousaf S. . Identification and clinical characterization of Hermansky-Pudlak syndrome alleles in the Pakistani population. Pigment Cell Melanoma Res, doi: 10.1111/pcmr.12438 (2015). PMC5062593 26575419

[b34] Bittles A. Consanguinity and its relevance to clinical genetics. Clin Genet 60, 89–98 (2001). 11553039 10.1034/j.1399-0004.2001.600201.x

[b35] Hussain R. & Bittles A. H. The prevalence and demographic characteristics of consanguineous marriages in Pakistan. J Biosoc Sci 30, 261–275 (1998). 9746828 10.1017/s0021932098002612

[b36] Sengupta M. . Comprehensive analysis of the molecular basis of oculocutaneous albinism in Indian patients lacking a mutation in the tyrosinase gene. Br J Dermatol 163, 487–494, doi: 10.1111/j.1365-2133.2010.09830.x (2010). 20426782

[b37] Hutton S. M. & Spritz R. A. Comprehensive analysis of oculocutaneous albinism among non-Hispanic caucasians shows that OCA1 is the most prevalent OCA type. J Invest Dermatol 128, 2442–2450, doi: 10.1038/jid.2008.109 (2008). 18463683 PMC3515683

[b38] Puri N. . Type 2 oculocutaneous albinism (OCA2) in Zimbabwe and Cameroon: distribution of the 2.7-kb deletion allele of the P gene. Hum Genet 100, 651–656 (1997). 9341887 10.1007/s004390050568

[b39] Giebel L. B., Tripathi R. K., King R. A. & Spritz R. A. A tyrosinase gene missense mutation in temperature-sensitive type I oculocutaneous albinism. A human homologue to the Siamese cat and the Himalayan mouse. J Clin Invest 87, 1119–1122, doi: 10.1172/JCI115075 (1991). 1900309 PMC329910

[b40] Kidson S. H. & Fabian B. C. The effect of temperature on tyrosinase activity in Himalayan mouse skin. J Exp Zool 215, 91–97, doi: 10.1002/jez.1402150111 (1981). 6785376

[b41] King R. A., Mentink M. M. & Oetting W. S. Non-random distribution of missense mutations within the human tyrosinase gene in type I (tyrosinase-related) oculocutaneous albinism. Mol Biol Med 8, 19–29 (1991). 1943686

[b42] King R. A. . Temperature-sensitive tyrosinase associated with peripheral pigmentation in oculocutaneous albinism. J Clin Invest 87, 1046–1053, doi: 10.1172/JCI115064 (1991). 1900307 PMC329899

[b43] Kwon B. S., Halaban R. & Chintamaneni C. Molecular basis of mouse Himalayan mutation. Biochem Biophys Res Commun 161, 252–260 (1989). 2567165 10.1016/0006-291x(89)91588-x

[b44] Toyofuku K., Wada I., Spritz R. A. & Hearing V. J. The molecular basis of oculocutaneous albinism type 1 (OCA1): sorting failure and degradation of mutant tyrosinases results in a lack of pigmentation. Biochem J 355, 259–269 (2001). 11284711 10.1042/0264-6021:3550259 PMC1221735

[b45] Grimberg J. . A simple and efficient non-organic procedure for the isolation of genomic DNA from blood. Nucleic Acids Res 17, 8390 (1989). 2813076 10.1093/nar/17.20.8390 PMC334995

[b46] McKenna A. . The Genome Analysis Toolkit: a MapReduce framework for analyzing next-generation DNA sequencing data. Genome Res 20, 1297–1303, doi: 10.1101/gr.107524.110 (2010). 20644199 PMC2928508

[b47] Li H. & Durbin R. Fast and accurate short read alignment with Burrows-Wheeler transform. Bioinformatics 25, 1754–1760, doi: 10.1093/bioinformatics/btp324 (2009). 19451168 PMC2705234

[b48] Kelley L. A. & Sternberg M. J. Protein structure prediction on the Web: a case study using the Phyre server. Nat Protoc 4, 363–371, doi: 10.1038/nprot.2009.2 (2009). 19247286

[b49] Ahmed Z. M. . Nonsyndromic recessive deafness DFNB18 and Usher syndrome type IC are allelic mutations of USHIC. Hum Genet 110, 527–531, doi: 10.1007/s00439-002-0732-4 (2002). 12107438

