# Supplementary material for: Molecular outcomes, clinical consequences, and genetic diagnosis of Oculocutaneous Albinism in Pakistani population
Source: Sci Rep. 2017 Mar 7;7:44185. doi: 10.1038/srep44185 (PMC5339803; doi:10.1038/srep44185)
Supplement: Supplementary Information [file srep44185-s1.pdf]

Molecular outcomes, clinical consequences, and genetic diagnosis of Oculocutaneous Albinism in Pakistani population

Mohsin Shahzad[1,*], Sairah Yousaf[1,2,*], Yar M. Waryah[3], Hadia Gul[4], Tasleem Kausar[2], Nabeela Tariq[2], Umair Mahmood[1], University of Washington Center for Mendelian Genomics, Muhammad Ali[5], Muzammil A. Khan[4], Ali M. Waryah[3], Rehan S. Shaikh[2], Saima Riazuddin[1] & Zubair M. Ahmed[1]

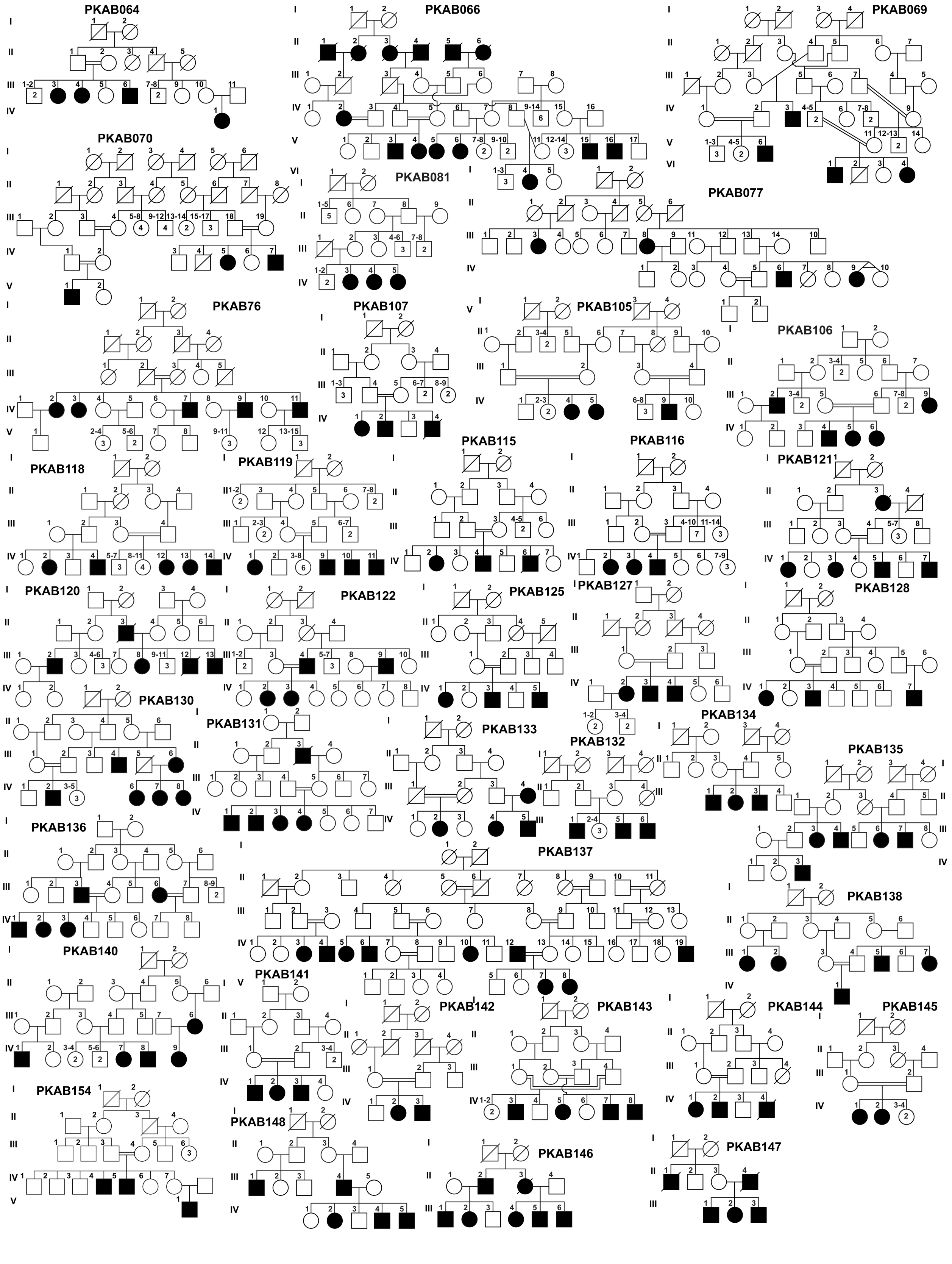
PKAB064
PKAB066
PKAB069
PKAB070
PKAB081
PKAB077
PKAB76
PKAB107
PKAB105
PKAB106
PKAB118
PKAB119
PKAB115
PKAB116
PKAB121
PKAB120
PKAB122
PKAB125
PKAB127
PKAB128
PKAB130
PKAB131
PKAB133
PKAB132
PKAB134
PKAB135
PKAB136
PKAB137
PKAB138
PKAB140
PKAB141
PKAB142
PKAB143
PKAB144
PKAB145
PKAB154
PKAB148
PKAB146
PKAB147

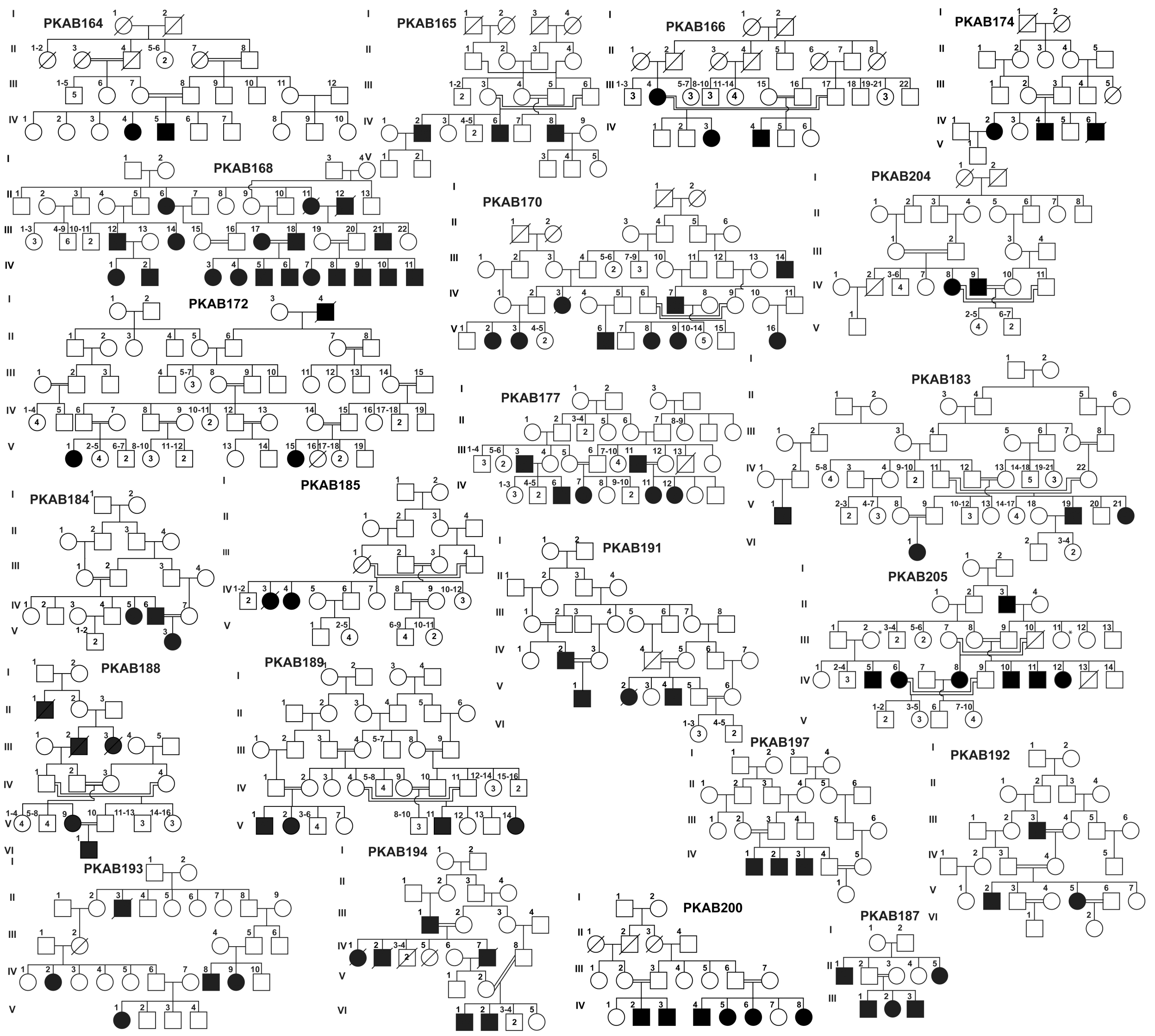
PKAB164
PKAB165
PKAB166
PKAB174
PKAB168
PKAB170
PKAB204
PKAB172
PKAB177
PKAB183
PKAB184
PKAB185
PKAB191
PKAB205
PKAB188
PKAB189
PKAB197
PKAB192
PKAB193
PKAB194
PKAB200
PKAB187

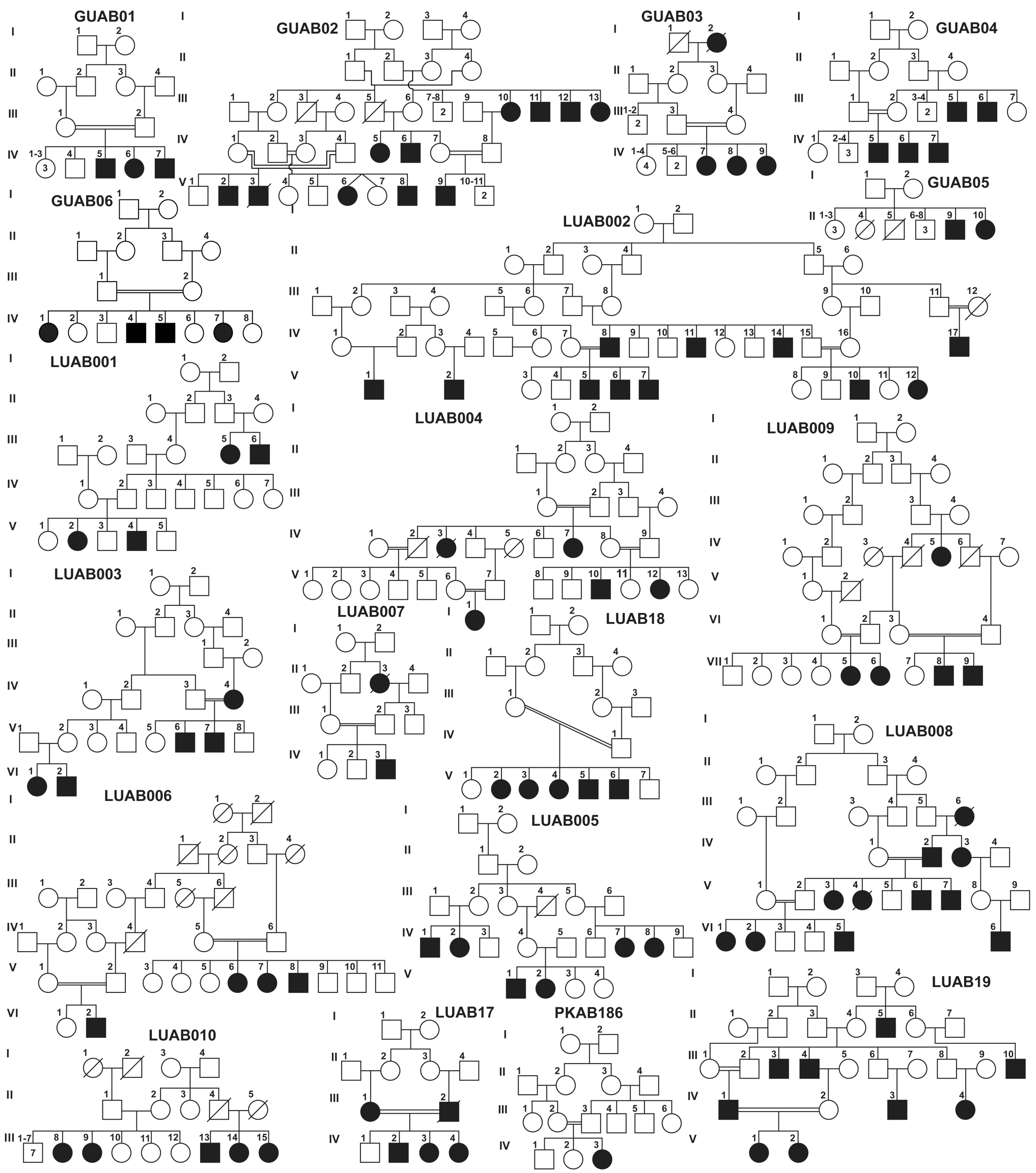

Supplementary Figure S1: Pedigrees of 80 families co-segregating OCA and mutations in OCA1-4 genes. Filled symbols represent individuals with nonsyndromic oculocutaneous albinism. Double line indicates consanguineous marriage.

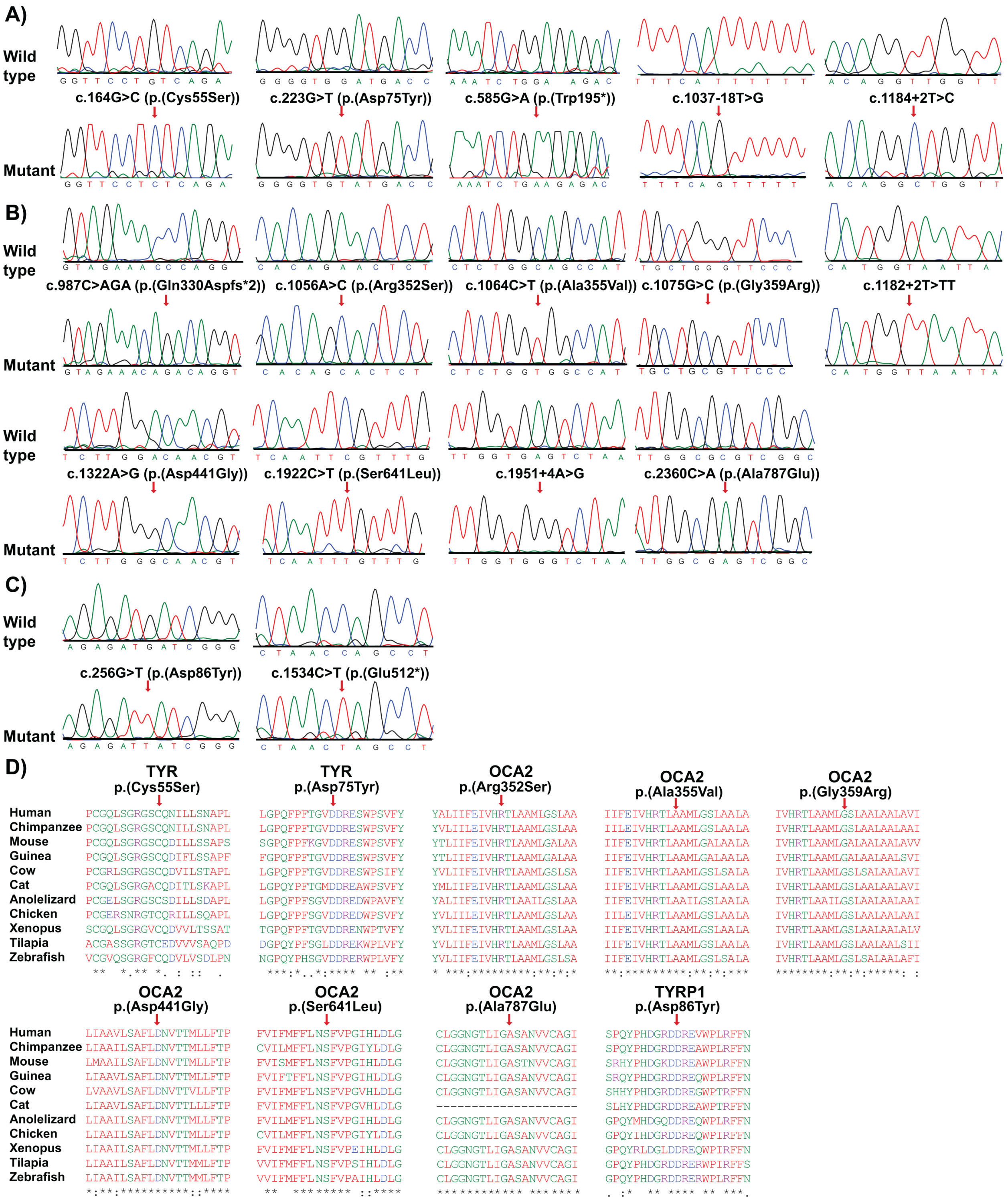

Supplementary Figure S2: Novel OCA genes alleles and evolutionary conservation of encoded proteins. (**A-C**) Nucleotide sequence chromatograms of exons of *TYR* (A), *OCA2* (**B**), and *TYRP1* (**C**) genes showing the wild type sequences and homozygosity for the mutation. (**D**) ClustalW alignments of TYR, OCA2 and TYRP1 proteins from various species revealed high conservation of amino acids mutated in our cohort.

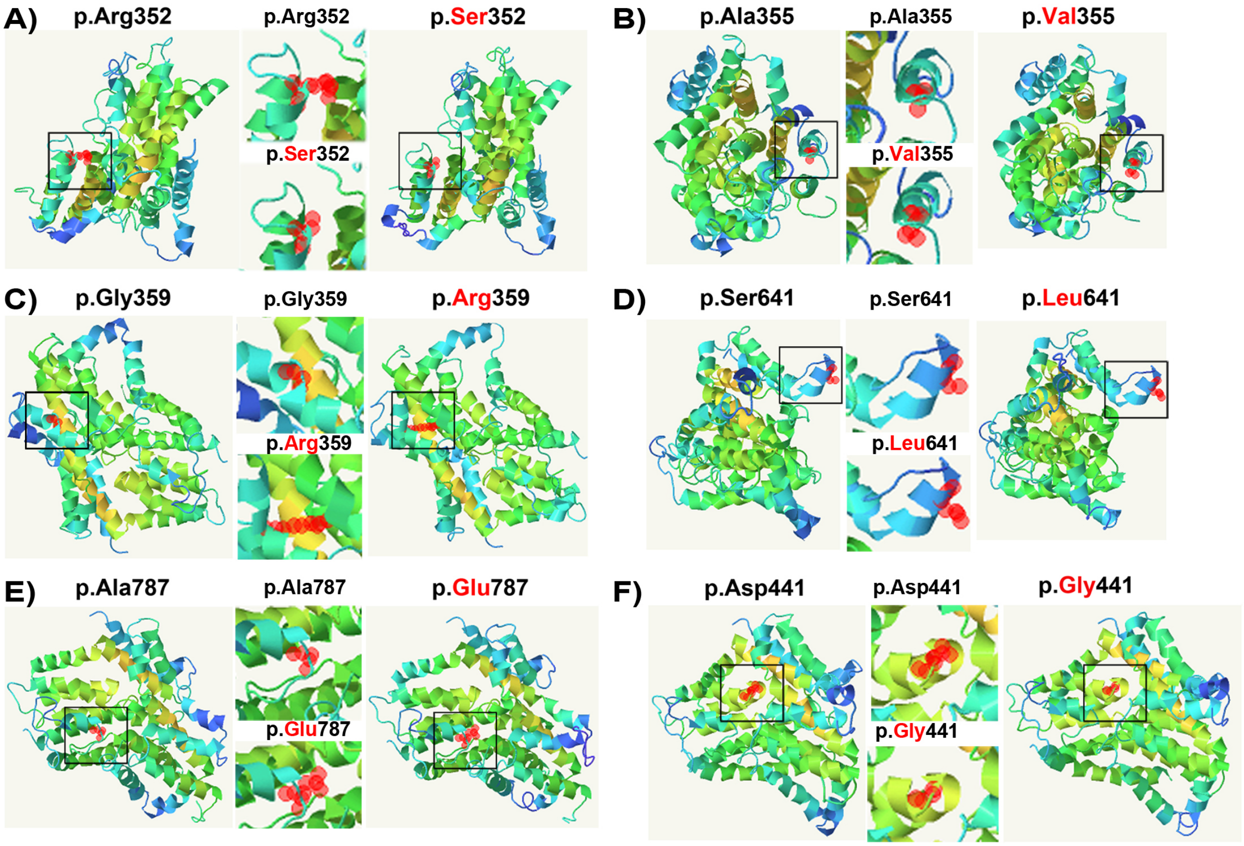

Supplementary Figure S3: Molecular modeling of OCA2 protein. Wild type and mutant residues are shown in red. Boxed regions are magnified to show the impact of the mutant alleles as compare to wild type residues on the structure of encoded protein.

(**A**) The p.(Arg352Ser) found in LUAB02, is predicted to replace the positively charged arginine with a small neutral serine, which not only will alter the interactional network of protein with other residues but leads to protein misfolding due to the change in hydrophobicity and subsequent loss of hydrogen bonds. (**B**) The p.Ala355 residue is part of transmembrane domain and replacing it with p.(Arg355Val) variant found in family PKAB136, which is known to be larger in size than alanine might lead to bumps and could affect the contacts with the lipid-membrane. (**C**) The p.(Gly359Arg) variant found in GUAB02 family, was predicted to cause the repulsion of ligands or other residues with the same charge and due to unusual torsion angles of arginine could also affects the contact with the lipid membrane. Only glycine is flexible enough to make these torsion angles, mutation into another residue will force the local backbone into an incorrect conformation and will disturb the local structure. (**D**) The p.(Ser641Leu) variant might cost the interaction with other proteins because of its bigger size. Leucine is more hydrophobic in nature, could result in loss of hydrogen bonds, which is predicted to disrupt the protein topology as well. (**E**) The p.(Ala787Glu) variant introduces a negative charge that can cause repulsion of ligands of same charge. Alanine is more hydrophobic than glutamic acid so the hydrophobic interactions, either in the core of the protein or on the surface, are predicted to be lost. (**F**) Finally, the molecular modeling of p.(Asp441Gly) variant found in LUAB06 suggested that mutant residue glycine is more hydrophobic, which could affect protein secondary structure due to the loss of hydrogen bonds and small size.

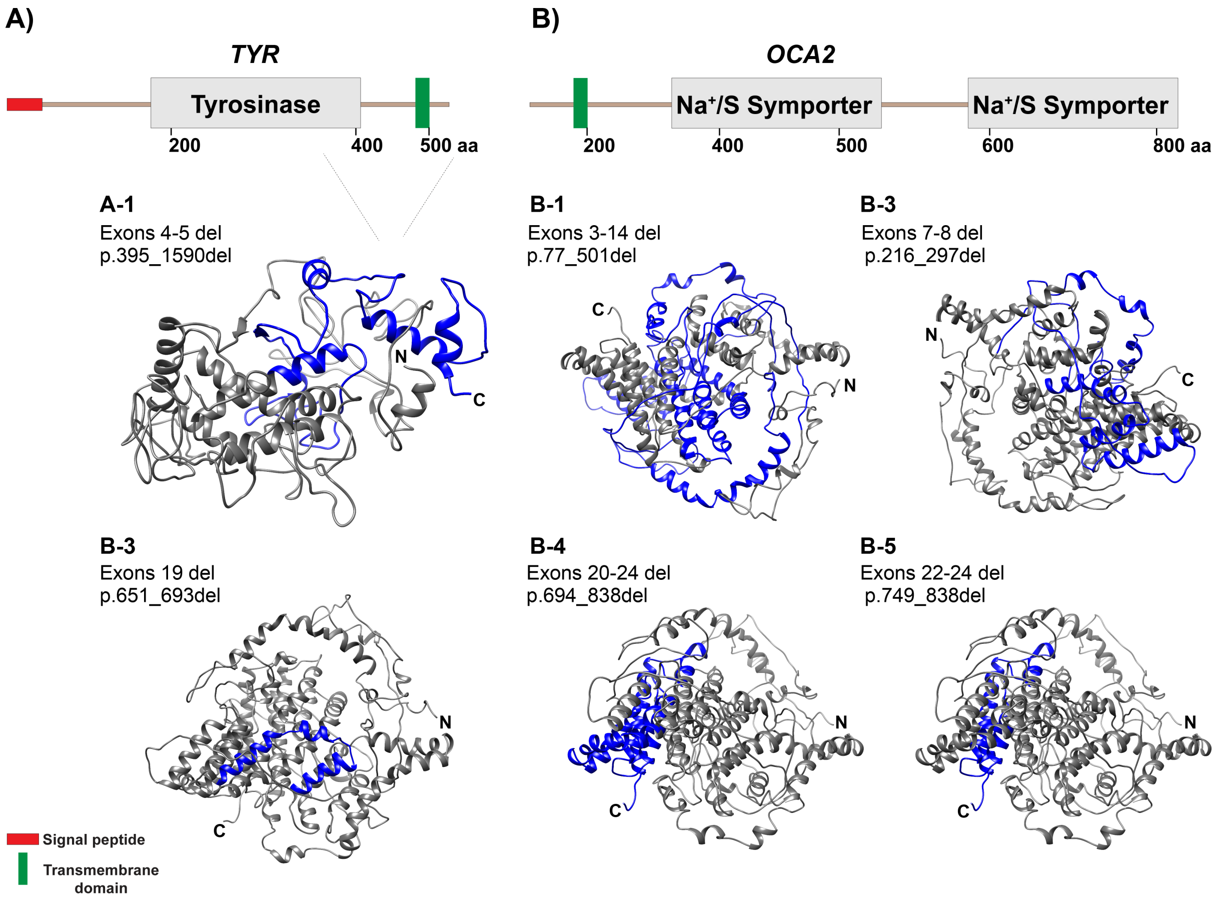

Supplementary Figure S4: Structural representation of gross deletions in TYR and OCA2 proteins. (**A**) TYR protein domain representation and its structural coverage. (**B**) The OCA2 protein domains and its predicted structure. Deleted residues are indicated in blue and wild type structure is presented in dark grey shade. S is for Sulphur in Na^{+}/S symporter domain.

TableS1. Pathogenic variants in OCA1-4 genes identified in Pakistani origins proband/families

Families	Mutation	Effect on protein	Allele frequency				Polyphen-2	Mutation Taster	SIFT	Reference
			PK	1000 genome	NHLBI6500	ExAC				
TYR										
PKAB74	c.62C>T	p.(Pro21Leu)	-	0	0	0	Probably damaging	Disease causing	Damaging	1
PKAB01, PKAB65	c.103T>C	p.(Cys35Arg)	-	0	0	0	Probably damaging	Disease causing	Damaging	1
Pakistani family	c.132T>A	p.(Ser44Arg)	-	0	0	0				2
PKAB115	c.164G>C	p.(Cys55Ser)	0/180	0	0	0.0000082	Probably damaging	Disease causing	Damaging	This study
PKAB174	c.223G>T	p.(Asp75Tyr)	0/176	0	0	0	Probably damaging	Disease causing	Damaging	This study
Pakistani family	c.230G>A	p.(Arg77Gln)		0.0004	0.0002	0.00009925	Probably damaging	Disease causing	Deleterious	2
Pakistani child	c.344_345delGA	p.(Arg115Thrfs*52)	-	0	0	0	-	-	-	3
Pakistani family	c.575C>A	p.(Ser192Tyr)		0.1234	0.2748	0.251800	Probably damaging	Polymorphism	Deleterious	2
PKAB200	c.585G>A	p.(Trp195*)	0/186	0	0.000153	0.0000082	-	-	-	This study
Pakistani family	c.593T>C	p.(Ile198Thr)	-	0	0	0.0000082	Probably damaging	Disease causing	Damaging	4
PKAB66, PKAB76, PKAB141, PKAB142, PKAB147	c.649C>T	p.(Arg217Trp)	-	0	0.000153	0.0001979	Probably damaging	Polymorphism	Tolerated	5
Pakistani family	c.715C>T	p.(Arg239Trp)	-	0	0	0.00004131	Probably damaging	Disease causing	Deleterious	2
Family1, PKAB57, PKAB118, PKAB120, PKAB143, PKAB145, PKAB165, PKAB185, PKAB189, PKAB194, LUAB05, LUAB08, LUAB19, PKAB204	c.832C>T	p.(Arg278*)	-	0	0	0.00019	-	-	-	6
PKAB128, PKAB154	c.896G>A	p.(Arg299His)	-	0	0	0.0000742	Probably damaging	Disease causing	Damaging	5
PKAB138, Family1	c.943-948delTCAGCT	p.(315-316delSerAla)	-	0	0	0	-	-	-	7
Pakistani proband	c.982G>C	p.(Glu328Gln)	-	0	0	0	Probably damaging	Disease causing	Damaging	6
PKAB105, PKAB183, PKAB186, PKAB188	c.1037-7T>A	Splicing error	-	0	0.001538	0	-	-	-	8
LUAB01	c.1037-18T>G	Splicing error	0/176	0	0	0.0000167	-	-	-	This study
PKAB197	c.1037G>A	p.(Gly346Glu)	-	0	0	0.0000082	Probably damaging	Disease causing	Damaging	3
Pakistani proband	c.1126C>T	p.(Gln376*)	-	0	0	0.0000082	-	-	-	6
PKAB119	c.1147G>A	p.(Asp383Asn)	-	0	0	0.000165	Probably damaging	Disease causing	Damaging	9
PKAB130	c.1184+2T>C	Splicing error	0/178	0	0	0.0000082	-	-	-	This study
PKAB193	c.1204C>T	p.(Arg402*)	-	0	0	0.00004984	-	-	-	10
PKAB153	c.1217C>T	p.(Pro406Leu)		0.002	0.00362	0	Probably damaging	Disease causing	Damaging	11
PKB103	c.1231T>C	p.(Tyr411His)	-	0		0	Probably damaging	Disease causing	Damaging	1
PKAB64, PKAB168	Exons 4-5 deletion	Frame shift	0/180	0	0	0	-	-	-	This study
Family10, Family 11, PKAB81, PKAB106, PKAB107, PKAB140, PKAB184, PKAB191, LUAB03, LUAB19, GUAB01	c.1255G>A	p.Gly419Arg)	-	0	0	0.00003312	Probably damaging	Disease causing	Damaging	12
Pakistani proband	c.1291C>A	p.(Pro431Thr)	-	0	0.000153	0	Probably damaging	Disease causing	Damaging	10
Pakistani proband	c.1292C>T	p.(Pro431Leu)	-	0	0	0	Probably damaging	Disease causing	Damaging	6
Pakistani proband	c.1357C>T	p.(Gln453*)	-	0	0	0	-	-	-	13
OCA2										
PKAB70	Exons 3-14 deletion	Frame shift	0/176	0	0	0	-	-	-	This study
PKAB69, PKAB77	Exons 7-8 deletion	Frame shift	0/180	0	0	0	-	-	-	This study
LUAB04	c.987C>AGA	p.(Gln330Aspfs*2)	0/184	0	0	0	-	-	-	This study
PKAB60, PKAB68, PKAB79, PKAB127, PKAB132, PKAB133, PKAB134, PKAB135, PKAB151, PKAB152, PKAB177, LUAB07, GUAB03	c.1045-15T>G	Splicing error	-	0	0	0.00004136	-	-	-	1
LUAB02	c.1056A>C	p.(Arg352Ser)	0/182	0	0	0	Probably damaging	Disease causing	Damaging	This study
PKAB136	c.1064C>T	p.(Ala355Val)	0/184	0.001	0	0.0002892	Probably damaging	Disease causing	Tolerated	This study
GUAB02	c.1075 G>C	p.(Gly359Arg)	0/176	0	0	0	Probably damaging	Disease causing	Damaging	This study
PKAB144	c.1182+2T>TT	Splicing error	0/180	0	0	0	-	-	-	This study
PKAB148	c.1211C>T	p.(Thr404Met)	-	0	0.00769	0.00007417	Probably damaging	Disease causing	Damaging	14

LUAB06	c.1322A>G	p.(Asp441Gly)	0/178	0	0.000153	0.00001674	Probably damaging	Disease causing	Tolerated	This study
PKAB52, PKAB54, PKAB55, PKAB67, PKAB101, PKAB172, PKAB187, GUAB06, LUAB17	c.1456G>T	p.(Asp486Tyr)	-	0	0	0.00001674	Probably damaging	Disease causing	Damaging	1
PKAB63	c.1580T>G	p.(Leu527Arg)	-	0	0	0.0000082	Probably damaging	Disease causing	Damaging	1
PKAB122	c.1922C>T	p.(Ser641Leu)	0/184	0	0	0	Probably damaging	Disease causing	Damaging	This study
LUAB10	c.1951+4A>G	Splicing error	0/182	0	0	0	-	-	-	This study
PKAB137	Exon 19 deletion	Frame shift	0/174	0	0	0	-	-	-	This study
Pakistani individuals	c.1960delG	p.(Ala654Leufs*8)	-	0	0	0	-	-	-	15
PKAB121	Exons 20-24 deletion	Frame shift	0/176	0	0	0	-	-	-	This study
PKAB116	Exons 21-24 deletion	Frame shift	0/180	0	0	0	-	-	-	This study
PKAB58, PKAB72, LUAB09	c.2228C>T	p.(Pro743Leu)	-	0	0	0.00009078	Probably damaging	Disease causing	Damaging	15
PKAB170	c.2360C>A	p.(Ala787Glu)	0/182	0	0	0	Probably damaging	Disease causing	Damaging	This study
PKAB125	c.2360C>T	p.(Ala787Val)	-	0	0	0.00002478	Probably damaging	Disease causing	Damaging	16
TYRP1										
PKAB166	c.256G>T	p.(Asp86Tyr)	0/180	0	0	0	Probably damaging	Disease causing	Damaging	This study
PKAB131	c.647_668del	P.(Glu216Glyfs*42)	-	0	0	0	-	-	-	17
PKAB164, PKAB192, PKAB205	c.1067G>A	p.(Arg356Gln)	-	0	0	0.00002550	Probably damaging	Disease causing	Damaging	18
Family 2	c.1120C>T	p.(Arg374*)	-	0	0	0.00002492				19
PKAB146	c.1534C>T	p.(Glu512*)	0/184	0	0	0.00001653	-	-	-	This study
SLC45A2										
PKAB53	c.251T>C	p.(Leu84Pro)	-	0	0	0	Probably damaging	Disease causing	Damaging	17
Pakistani family	c.755G>A	p.(Gln272Lys)	-	0	0	0				2
PKAB59	c.889-6T>G	Splicing error	-	0	0	0	-	-	-	17
PKAB51, GUAB04, GUAB05	c.1532C>T	p.(Ala511Val)	-	0	0	0.0000082	Probably damaging	Disease causing	Damaging	17

PK: Pakistani controls

Table S2: Non-pathogenic variants observed in Pakistani OCA families

No.	Nucleotide change	Amino acid change	Location	dbSNP ID	No. of families
TYR (NM_000372)					
1	c.575C>A	p.(Ser192Tyr)	Exonic	*rs1042602*	16
2	c.1184+27C>T	-	Intronic	*rs147210895*	01
3	c.1184+50G>A	-	Intronic	*rs3793975*	08
4	c.1205G>A	p.(Arg402Gln)	Exonic	*rs147574809*	11
5	c.1412C>G	p.(Ala471Gly)	Exonic	*rs3913544*	17
6	c.1413G>A	p.(Ala471Ala))	Exonic	-	16
7	c.1446G>C	p.(Ala482Ala)	Exonic	*rs138173955*	18
OCA2 (NM_000275)					
8	c.-13 C>G	-	5' UTR	Novel variants	01
9	c.987C>A	p.(Thr329Thr)	Exonic	-	01
10	c.913G>A	p.(Arg305Trp)	Exonic	*rs1800401*	20
11	c.954G>A	p.(Met318Ile)	Exonic	*rs529219961*	01
12	c.1065G>A	p.(Ala355Ala)	Exonic	*rs1800404*	23
13	c.1183-4A>G	-	Intronic	*rs10852218*	30
14	c.1364+26A>G	-	Intronic	*rs1800410*	06
15	c.1365-15C>T	-	Intronic	*rs12910433*	07
16	c.1551C>T	p.(Cys517Cys)	Exonic	*rs1800411*	18
17	c.1636+78T>C	-	Intronic	*rs4778218*	12
18	c.1785-47A>G	-	Intronic	*rs7170989*	09
19	c.1951+45G>A/C	-	Intronic	*rs17566952*	02
20	c.2244+25C>G	-	Intronic	*rs7175046*	13
21	c.2328T>C	p.(Ala776Ala)	Exonic	*rs1800419*	41
22	c.2339G>A	p.(Gly780Asp)	Exonic	*rs141949212*	08
23	c.2338+75T>C	-	Intronic	*rs8025804*	12
24	c.2364G>A	p.(Ser788Ser)	Exonic	*rs12592307*	19
TYRP1 (NM_000550)					
25	c.259C>A	p.(Arg87Arg)	Exonic	*rs34509359*	01
26	c.729T>C	p.(Ser243Ser)	Exonic	*rs35866166*	02
27	c.1589A>G	p.(Gln530Arg)	Exonic	*rs41305645*	01
SLC45A2 (NM_016180)					
28	c.814G>A	p.(Glu272Lys)	Exonic	*rs26722*	23
29	c.987A>G	p.(Thr329Thr)	Exonic	*rs2287949*	12
30	c.1122C>G	p. (Phe374Phe)	Exonic	*rs16891982*	11
31	c.1122C>G	p.(Phe374Leu)	Exonic	*rs16891982*	20
32	c.1155T>A	p.(Ser385Ser)	Exonic	-	01

Table S3: Prevalence of OCA1 and OCA2 in different populations

Population	Sample size	% Prevalence		Prevalent Alleles		Reference
		OCA1	***OCA2***	***TYR***	***OCA2***	
**Pakistan**	143 families	37.06	31.46	c.832C>T<br>c.1255G>A	c.1045-15T>G	This study; 1
**Chinese**	51 patients	76.40	11.70	c.929dupC	c.406C>T	20
	52 patients	50	15.40	c.896G>A	NA	21
	127 patients	70.10	10.20	c.896G>A	c.1363A>G	22
**Indian**	82 patients	59.80	10.90	NA	c.2359G>A	23
	23 families	17.39	8.69	NA	c.1453G>A	24
	14 families	100	NA	c.832C>T	NA	25
**American**	36 patients	56	8	c.1205G>A	NA	26
	121 patients	69	18	c.1118C>A	c.1327G>A	26
**European**	63 patients	46	29	c.1205G>A	Exon 3-20 deletion	27
**Italian**	45 patients	73.30	13.30	c.1205G>A	c.2216T>C	28
	279 patients	57.70	17.92	c.230G>A<br>c.1037-7T>A<br>c.1118C>A	c.913C>T<br>c.1327G>A	29
**African**	186 families	NA	82	NA	2.7kb del	30
	111 patients	NA	77	NA	2.7kb del	31
	5 families	NA	100	NA	2.7kb del	32
**Japanese**	NA	34	<10	c.929dupC	c.1182G>A	33,34
	9 families	55.50	NA	c.929dupC	NA	8
	16 patients	100	NA	c.929dupC	NA	35
	26 patients	100	NA	c.929dupC	NA	36
**Korean**	12 patients	66.7	NA	c.929dupC	NA	37
	21 patients	66.70	5	c.929dupC	NA	38
	12 patients	50	NA	c.929dupC	NA	39

NA: Not available

Table S4: Primers used for the mutational analysis of *OCA1-4* genes

Gene	Forward Primer	Reverse Primer	Product Size (bp)
TYR_Ex1a	AGAGAAATCTGTGACTCCAATTAGC	CATCCAGACAAAGAGGTCATAAATA	597
TYR_Ex1b	GTGAGAAGAAACATCTTCGATTTG	CCCTACTCTGACATCGTATATCTAGC	500
TYR_Ex2	ACAATTTGTTTAACATGAGGGTGTT	ACCTCCTAGGACTTTGGATAAGAGA	326
TYR_Ex3	TCACATAGGTTTTCAGTCATTAAAGT	AAATCCAATGAGCACGTTATTTAT	250
TYR_Ex4	TGTTTCTTAGTCTGAATAACCTTTTCC	CAGCAATTCCTCTGAAAGAAAGTAA	248
TYR_Ex5	TCGTAACAATGGTGGTAACAATA	GGCCCTACTCTATTGCCTAAG	300
OCA2_Ex2	CTTTCATGAAGAGTGGTTTCTTTCT	CTCATGGAAACCCAATCTGTG	345
OCA2_Ex3	CATTATTTCTGTGTTGGTGATTCTG	GTGCAATGCTCAGAAACTCTTACTT	196
OCA2_Ex4	AGAGATAACCACACCTCTCTTGCTT	AAAGATGGAGGGGCCATGTA	294
OCA2_Ex5	ATGCTTTGAGATGGAAGTTACTCAA	CTATACAGCCAAAGGCACACAG	228
OCA2_Ex6	CTTACTGCTCTCATACCACACCTCT	CAAGTGTCTCCTTGTGTTTCAGAT	191
OCA2_Ex7	CTTGTGGTTCTCCTTGACACTCT	AAATGAGATTTCACAATTCCTTTCA	233
OCA2_Ex8	GAAACAAATACCTAGACCGAGCAGT	CCAACACCTCACTCACTGAGAACT	199
OCA2_Ex9	CTTGTGAACAGTAAGGTCGTTGTTT	ATCTCAAGCCTCCCTGACTGT	248
OCA2_Ex10	TGTGGTACACAGTGGCAGATATAGA	GAATCCTGGAACATCTTTGAGC	250
OCA2_Ex11	ATTAATTGGCAAATTTGTGCTTTG	CGCTGTGTCTTTAACATAATGAAGG	200
OCA2_Ex12	GAGCTCAAATGTGTAAGGGATCAT	TCAGGATAGAATTATTAAATGCAACATC	182
OCA2_Ex13	TAATGAAAGGCTGCCTCTGTTCTAC	TTCATGCACCTGAGAATGGAAC	298
OCA2_Ex14	GGACTACTTTCATTTTCCTCCATTT	AGAGCTCTAACTAAGTGGAGGTGTG	233
OCA2_Ex15	CCATTTCATTTCCCCTTGTTTAT	CCTACATGAGGTTGCACTTGTACT	198
OCA2_Ex16	TGATATCTGAGGTCATGGGAGAC	ATGTTCTGCTGCACACCAAG	247
OCA2_Ex17	GACCAGGGAAGTAATGAGTCTCTTC	AAAGGCATCACTCACTCTCTTCTT	163
OCA2_Ex18	GAGTAAATGAGCTGTGGTTTCTCTC	CAGAATGTGACAAAGCCTATGAAC	184
OCA2_Ex19	ATCGGTGTGTTAACAGTGGAACTAT	TTCACCAAATAAAACATGAAATACAAA	250
OCA2_Ex20	GGTTCTAAACTGATTCTCACCACAC	TAATTAATGGGACCTGTTCTTACCA	204
OCA2_Ex21	ACTGCAGCTGCTATTGTCCTC	GGCTATGTCCAGGCTAAAGTTG	184
OCA2_Ex22	ATTTGGATTTTCTAAATTGGTCACA	CTAACTGTTGCTTTGGGCTGA	250
OCA2_Ex23	ATTACAAACCAAAGAGAACAGAAGC	CAGTTTAACAGAAAATTTAAAGGGAAT	203
OCA2_Ex24	CAGATGCCATAACTCATTTCTCTTT	AAGTTTCCTTTAGTCTTCGAGCAAT	157
TYRP1.Ex2	GGGCATACCATTTTAAGTACCAAG	TTGAGTCTCATGCAGGACTTATG	579
TYRP1.Ex3	TGATTATGCTCTTTCTCTACCCATC	AAAATCACTTACAAACAAGGCATCT	459
TYRP1.Ex4	TGTCAGAGAGTAGACCAAACAGAAA	CCTTTCAGGCAACATATTTAATCTC	354
TYRP1.Ex5	TTTAAAGAGCGACAATAAGAACTCC	ATCTCATTCACATAAACACACAGGA	361
TYRP1.Ex6	ATCATTGCTATTACCTGGAAAAGTG	TGCTCAGCTTGAACTGATAACTATG	332
TYRP1.Ex7	GAATATTGGATGCCTTTAGAACTCA	AAAGATAACACATTTGCTTTTGGAA	292
TYRP1.Ex8	ATCTGTCCACTTTTTGGTGATAACT	GCATAAGAGAGTAGGGCATTTGTTA	297
SLC45A2.Ex1	GTCAAATCCAGTTTGAAACACAGAC	TGCAGAGGTACACACTAAGACACAT	524
SLC45A2.Ex2	ATTACAAAACGCGGATGATTCTAA	TTAGTGGAAGTGCCTCATTGTCT	300
SLC45A2.Ex3	GAGTGTCTATGCATGAGGAAAATG	ACTCTTCTCGTCAAACAGACAAAAC	499
SLC45A2.Ex4	ATACATAACATGTCTGTGTGTTCTGG	ACAGGTGTTAATGGAGGAAATGAT	293
SLC45A2.Ex5	AGAGTGCATGAGAAGGGTTCTTAC	CCAAGTTGTGCTAGACCAGAAAC	250
SLC45A2.Ex6	TCAGAAGAAAGGATTGTCTGAAATTA	CTTATTTTCCAGCTCTGCTCTACAC	391
SLC45A2.Ex7	ATTTACTATCTGGCTGGTCACAGAA	AGAAAATGTTAACTTCCTGCCATGT	390

Table S5: Primers used for tetra primers ARMS assay

Gene	Forward Primer	Reverse Primer	Product Size (bp)
TYR: c.649C>T			
	Forward inner primer (C allele)	CCTTGGCATAGACTCTTCTTGTGGC	191
	Reverse inner primer (T allele)	GCTTCTGGATTTCTTGTTCCCAACA	129
	Forward outer primer	ATGTGTCAATGGATGCACTGCTTG	270
	Reverse outer primer	AAGAGGAGAAGAATGATGCTGGGC	
TYR: c.832C>T			
	Forward inner primer (C allele)	GAGGGTGTTTTGTACAGATTGTCTGTATCC	170
	Reverse inner primer (T allele)	TGATGGCTGTTGTACTCCTCCAAGCA	100
	Forward outer primer	CGATAATTAGGAGTTCCAACATTTCTGCC	269
	Reverse outer primer	TTATCCATGGAACCAGATTCATATTGGG	
OCA2: c.1045-15 T>G			
	Forward inner primer (G allele)	ACTGGAACGCGGTAATTTTCG	204
	Reverse inner primer (T allele)	ACGATCTGGAAAGAAGCGCA	298
	Forward outer primer	GACAGTTTGCTATGGCCTTTCTCA	461
	Reverse outer primer	ATAGTGAAAAAACCAGCGAAAGCC	
OCA2: c.1456G>T			
	Forward inner primer (T allele)	AGCTGCCACTGCCATCGAGT	192
	Reverse inner primer (G allele)	TGGAAACAATAATGACATTTGGAGGATC	137
	Forward outer primer	TTCCTCCATTTGTGACAGGTTGTG	281
	Reverse outer primer	GCACTTACTGTGAAGAGGTGGCGT	
OCA2: c.2228C>T			
	Forward inner primer (C allele)	GCGTCGTCCCTGATTGACAACATACC	168
	Reverse inner primer (T allele)	GCAACTCACCATGGTAGCAGTGACCA	250
	Forward outer primer	GCTTGCACACCTATGTCTGCCTTGGT	378
	Reverse outer primer	TTCGTCCTCTACACCTGTGAGTGCAGC	
SLC45A2: c.1532C>T			
	Forward inner primer (T allele)	GACCGTTGTCGTCGTGGTGATCACCGT	185
	Reverse inner primer (C allele)	AACAGCCTATCAGTGCCACCGCAGCCG	140
	Forward outer primer	CCACCCTCACATGCATGGTGCAGCTG	272
	Reverse outer primer	AGTGGGCGTCCAATGGGCAGACCCTAAG	

Supplementary References

1 Jaworek, T. J. *et al.* Molecular genetic studies and delineation of the oculocutaneous albinism phenotype in the Pakistani population. *Orphanet J Rare Dis* **7**, 44, doi:10.1186/1750-1172-7-44 (2012).

2 Shah, S. A. *et al.* Mutational spectrum of the TYR and SLC45A2 genes in Pakistani families with oculocutaneous albinism, and potential founder effect of missense substitution (p.Arg77Gln) of tyrosinase. *Clin Exp Dermatol* **40**, 774-780, doi:10.1111/ced.12612 (2015).

3 Oetting, W. S., Fryer, J. P. & King, R. A. A dinucleotide deletion (-delta GA115) in the tyrosinase gene responsible for type I-A (tyrosinase negative) oculocutaneous albinism in a Pakistani individual. *Hum Mol Genet* **2**, 1047-1048 (1993).

4 Shah, S. A. *et al.* Identification of a novel mutation (p.Ile198Thr) in gene TYR in a Pakistani family with nonsyndromic oculocutaneous albinism. *Clin Exp Dermatol* **39**, 646-648, doi:10.1111/ced.12382 (2014).

5 Tripathi, R. K., Strunk, K. M., Giebel, L. B., Weleber, R. G. & Spritz, R. A. Tyrosinase gene mutations in type I (tyrosinase-deficient) oculocutaneous albinism define two clusters of missense substitutions. *Am J Med Genet* **43**, 865-871, doi:10.1002/ajmg.1320430523 (1992).

6 Tripathi, R. K. *et al.* Mutations of the tyrosinase gene in Indo-Pakistani patients with type I (tyrosinase-deficient) oculocutaneous albinism (OCA). *Am J Hum Genet* **53**, 1173-1179 (1993).

7 King, R. A. *et al.* Tyrosinase gene mutations in oculocutaneous albinism 1 (OCA1): definition of the phenotype. *Hum Genet* **113**, 502-513, doi:10.1007/s00439-003-0998-1 (2003).

8 Goto, M. *et al.* Tyrosinase gene analysis in Japanese patients with oculocutaneous albinism. *J Dermatol Sci* **35**, 215-220, doi:10.1016/j.jdermsci.2004.06.007 (2004).

9 Spritz, R. A., Strunk, K. M., Giebel, L. B. & King, R. A. Detection of mutations in the tyrosinase gene in a patient with type IA oculocutaneous albinism. *N Engl J Med* **322**, 1724-1728, doi:10.1056/NEJM199006143222407 (1990).

10 Spritz, R. A. Molecular genetics of oculocutaneous albinism. *Semin Dermatol* **12**, 167-172 (1993).

11 Giebel, L. B. *et al.* Tyrosinase gene mutations associated with type IB ("yellow") oculocutaneous albinism. *Am J Hum Genet* **48**, 1159-1167 (1991).

12 King, R. A., Mentink, M. M. & Oetting, W. S. Non-random distribution of missense mutations within the human tyrosinase gene in type I (tyrosinase-related) oculocutaneous albinism. *Mol Biol Med* **8**, 19-29 (1991).

13 Oetting, W. S. & King, R. A. Molecular analysis of type I-A (tyrosinase negative) oculocutaneous albinism. *Hum Genet* **90**, 258-262 (1992).

14 Spritz, R. A., Fukai, K., Holmes, S. A. & Luande, J. Frequent intragenic deletion of the P gene in Tanzanian patients with type II oculocutaneous albinism (OCA2). *Am J Hum Genet* **56**, 1320-1323 (1995).

15 Lee, S. T. *et al.* Mutations of the P gene in oculocutaneous albinism, ocular albinism, and Prader-Willi syndrome plus albinism. *N Engl J Med* **330**, 529-534, doi:10.1056/NEJM199402243300803 (1994).

16 Oetting, W. S. *et al.* Mutations of the human P gene associated with Type II oculocutaneous albinism (OCA2). Mutations in brief no. 205. Online. *Hum Mutat* **12**, 434, doi:10.1002/(SICI)1098-1004(1998)12:6<434::AID-HUMU16>3.0.CO;2-7 (1998).
17 Kausar, T. *et al.* Genetic studies of TYRP1 and SLC45A2 in Pakistani patients with nonsyndromic oculocutaneous albinism. *J Invest Dermatol* **133**, 1099-1102, doi:10.1038/jid.2012.432 (2013).
18 Rooryck, C., Roudaut, C., Robine, E., Musebeck, J. & Arveiler, B. Oculocutaneous albinism with TYRP1 gene mutations in a Caucasian patient. *Pigment Cell Res* **19**, 239-242, doi:10.1111/j.1600-0749.2006.00298.x (2006).
19 Forshew, T. *et al.* Identification of novel TYR and TYRP1 mutations in oculocutaneous albinism. *Clin Genet* **68**, 182-184, doi:10.1111/j.1399-0004.2005.00460.x (2005).
20 Wei, A. H., Zang, D. J., Zhang, Z., Yang, X. M. & Li, W. Prenatal genotyping of four common oculocutaneous albinism genes in 51 Chinese families. *J Genet Genomics* **42**, 279-286, doi:10.1016/j.jgg.2015.05.001 (2015).
21 Wei, A., Yang, X., Lian, S. & Li, W. Implementation of an optimized strategy for genetic testing of the Chinese patients with oculocutaneous albinism. *J Dermatol Sci* **62**, 124-127, doi:10.1016/j.jdermsci.2011.02.009 (2011).
22 Wei, A. *et al.* A comprehensive analysis reveals mutational spectra and common alleles in Chinese patients with oculocutaneous albinism. *J Invest Dermatol* **130**, 716-724, doi:10.1038/jid.2009.339 (2010).
23 Sengupta, M. *et al.* Comprehensive analysis of the molecular basis of oculocutaneous albinism in Indian patients lacking a mutation in the tyrosinase gene. *Br J Dermatol* **163**, 487-494, doi:10.1111/j.1365-2133.2010.09830.x (2010).
24 Renugadevi, K., Sil, A. K., Perumalsamy, V. & Sundaresan, P. Spectrum of candidate gene mutations associated with Indian familial oculocutaneous and ocular albinism. *Mol Vis* **16**, 1514-1524 (2010).
25 Chaki, M. *et al.* Higher prevalence of OCA1 in an ethnic group of eastern India is due to a founder mutation in the tyrosinase gene. *Mol Vis* **11**, 531-534 (2005).
26 Hutton, S. M. & Spritz, R. A. Comprehensive analysis of oculocutaneous albinism among non-Hispanic caucasians shows that OCA1 is the most prevalent OCA type. *J Invest Dermatol* **128**, 2442-2450, doi:10.1038/jid.2008.109 (2008).
27 Rooryck, C. *et al.* Molecular diagnosis of oculocutaneous albinism: new mutations in the OCA1-4 genes and practical aspects. *Pigment Cell Melanoma Res* **21**, 583-587, doi:10.1111/j.1755-148X.2008.00496.x (2008).
28 Gargiulo, A. *et al.* Molecular and clinical characterization of albinism in a large cohort of Italian patients. *Invest Ophthalmol Vis Sci* **52**, 1281-1289, doi:10.1167/iovs.10-6091 (2011).
29 Mauri, L. *et al.* Clinical evaluation and molecular screening of a large consecutive series of albino patients. *J Hum Genet*, doi:10.1038/jhg.2016.123 (2016).
30 Puri, N. *et al.* Type 2 oculocutaneous albinism (OCA2) in Zimbabwe and Cameroon: distribution of the 2.7-kb deletion allele of the P gene. *Hum Genet* **100**, 651-656 (1997).

31 Stevens, G., Ramsay, M. & Jenkins, T. Oculocutaneous albinism (OCA2) in sub-Saharan Africa: distribution of the common 2.7-kb P gene deletion mutation. *Hum Genet* **99**, 523-527 (1997).
32 Lund, P. M., Puri, N., Durham-Pierre, D., King, R. A. & Brilliant, M. H. Oculocutaneous albinism in an isolated Tonga community in Zimbabwe. *J Med Genet* **34**, 733-735 (1997).
33 Suzuki, T. & Tomita, Y. Recent advances in genetic analyses of oculocutaneous albinism types 2 and 4. *J Dermatol Sci* **51**, 1-9, doi:10.1016/j.jdermsci.2007.12.008 (2008).
34 Suzuki, T. *et al.* Six novel P gene mutations and oculocutaneous albinism type 2 frequency in Japanese albino patients. *J Invest Dermatol* **120**, 781-783, doi:10.1046/j.1523-1747.2003.12127.x (2003).
35 Tanita, M. *et al.* Polymorphic sequences of the tyrosinase gene: allele analysis on 16 OCA1 patients in Japan indicate that three polymorphic sequences in the tyrosinase gene promoter could be powerful markers for indirect gene diagnosis. *J Hum Genet* **47**, 1-6, doi:10.1007/s10038-002-8648-3 (2002).
36 Tomita, Y., Miyamura, Y., Kono, M., Nakamura, R. & Matsunaga, J. Molecular bases of congenital hypopigmentary disorders in humans and oculocutaneous albinism 1 in Japan. *Pigment Cell Res* **13 Suppl 8**, 130-134 (2000).
37 Ko, J. M., Yang, J. A., Jeong, S. Y. & Kim, H. J. Mutation spectrum of the TYR and SLC45A2 genes in patients with oculocutaneous albinism. *Mol Med Rep* **5**, 943-948, doi:10.3892/mmr.2012.764 (2012).
38 Park, S. H., Chae, H., Kim, Y. & Kim, M. Molecular analysis of Korean patients with oculocutaneous albinism. *Jpn J Ophthalmol* **56**, 98-103, doi:10.1007/s10384-011-0098-z (2012).
39 Park, S. K. *et al.* Prevalent and novel mutations of the tyrosinase gene in Korean patients with tyrosinase-deficient oculocutaneous albinism. *Mol Cells* **7**, 187-191 (1997).
